# Long-Term Cigarette Smoke Exposure Promotes Neutrophil Ferroptosis Resistance, Inducing Neutrophil Extracellular Trap Formation and Driving Glucocorticoid Resistance in Chronic Obstructive Pulmonary Disease

**DOI:** 10.34133/research.0751

**Published:** 2025-07-15

**Authors:** Lu Wang, Lijie Zhu, Ying Tang, Zhongmei Wen, Liping Peng, Xinxin Ci

**Affiliations:** ^1^ Department of Respiratory Medicine, The First Hospital of Jilin University, Changchun 130021, China.; ^2^ Institute of Translational Medicine, The First Hospital of Jilin University, Changchun 130001, China.

## Abstract

Glucocorticoid resistance increases the frequency of acute exacerbations and the risk of death in chronic obstructive pulmonary disease (COPD) patients with a history of long-term heavy smoking. In this study we aimed to investigate the role of neutrophil ferroptosis resistance and the formation of neutrophil extracellular traps (NETs) in cigarette smoke (CS)-induced glucocorticoid resistance in COPD. We collected clinical specimens from COPD patients and healthy subjects. A mouse model of COPD induced by CS exposure was established in vivo. Neutrophils were isolated from the peripheral blood of human donors and exposed to CS extract in vitro. We found extensive NET formation was observed in COPD patients with a history of long-term heavy smoking and was closely related to glucocorticoid resistance. In vivo, we found that prolonged CS exposure promoted NET formation and that rendered dexamethasone (Dex) treatment ineffective at alleviating lung inflammation in COPD model mice. However, the NET degrading agent deoxyribonuclease I could increase sensitivity to Dex in COPD model mice. In vitro experiments demonstrated that CS extract increased neutrophil cell viability by activating the Nrf2/SLC7A11/GPX4 pathway and inducing ferroptosis resistance in neutrophils. And we found that neutrophil specific GPX4 knockout inhibited CS-induced NET formation, increased sensitivity to Dex, and alleviated CS-induced glucocorticoid resistance in vivo and in vitro. In conclusion CS promotes glucocorticoid resistance in COPD by inducing ferroptosis resistance in neutrophils, further resulting in NET formation.

## Introduction

Chronic obstructive pulmonary disease (COPD) is a persistent respiratory condition characterized by continuous airflow limitation. As the third leading cause of death globally, it affects approximately 400 million individuals [1]. Treatment outcomes for COPD remain poor, primarily because some long-term heavy smokers in Global Initiative for Chronic Obstructive Lung Disease (GOLD) groups C and D exhibit resistance to glucocorticoids [2,3]. Glucocorticoids exert strong anti-inflammatory effects by activating glucocorticoid receptor α (GRα) and increasing the expression of histone deacetylase 2 (HDAC2) [4,5]. This process leads to histone deacetylation, which inhibits the transcription of proinflammatory genes (such as IL-8, IL-1β, IL-6, and TNFα) while promoting the expression of anti-inflammatory genes [6–8]. However, in clinical practice, a subset of COPD patients do not respond adequately to standard doses of glucocorticoids. These patients are predominantly those with a history of long-term heavy smoking, exhibit relatively high mortality rates, and require substantial clinical resources [3,9,10].

Cigarette smoking, the most common etiological factor of COPD, leads to neutrophil infiltration and the formation of neutrophil extracellular traps (NETs) in the airways of COPD patients [11,12]. Neutrophils, the primary inflammatory cells in COPD, can be activated by cigarette smoke extract (CSE) to facilitate NET formation [13,14]. NETs are weblike structures released by neutrophils upon stimulation and are composed primarily of double-stranded DNA, histones, and granular proteins such as neutrophil elastase (NE), myeloperoxidase (MPO), and tissue factors. NE constitutes one-third of the cytoplasmic proteins in NETs and is often used as an indirect indicator of the degree of NET release [15,16]. In asthma models, NETs have been shown to participate in glucocorticoid resistance by promoting the secretion of inflammatory factors such as IL-8 and IL-17, preventing dexamethasone (Dex) from alleviating airflow limitation and airway obstruction [17,18]. However, the contribution of NETs to glucocorticoid resistance in COPD is still unclear.

There are 2 forms of NETosis: suicidal lytic NETosis and vital NETosis [19,20]. While the classic NET inducer phorbol 12-myristate 13-acetate (PMA) promotes reactive oxygen species (ROS) generation and subsequently induces NET formation through the lysis of neutrophils [21], CSE induces NET formation through the activation of AKT and peptidylarginine deiminase 4 (PADI4), and this type of NETosis is a form of vital NETosis that is independent of the ROS system [13,22,23]. In vital NETosis, the release of NETs does not disrupt the plasma membrane; rather, it involves the secretion of protein-coated chromatin through vesicles, enabling neutrophils to survive and carry out additional functions [19]. The production of NETs, particularly the formation of vital NETosis, requires the activation and recruitment of numerous viable neutrophils to exert continuous biological effects [20,24]. However, neutrophils are short-lived cells, with a half-life of approximately 24 h in tissues [25]. Therefore, the prolonged recruitment of neutrophils and the release of NETs in the airways of COPD patients are inevitably due to neutrophils developing resistance to a specific form of cell death following exposure to cigarette smoke (CS). Mechanically, CS can induce ferroptosis in airway epithelial cells [26]; however, its effects on neutrophils have not been thoroughly investigated. In inflammatory cells, resistance to ferroptosis leads to the sustained activation of neutrophils and increased proinflammatory activity, thereby exacerbating the negative effects of inflammation on the body. It is worth investigating whether CS-induced NET formation is related to neutrophil ferroptosis resistance and how abnormal NET formation is associated with glucocorticoid resistance in COPD.

In vitro studies have demonstrated that CSE can sustain neutrophil activity by promoting AKT phosphorylation and inhibiting the production of ROS [13,23]. Ferroptosis, which is also closely associated with ROS, is characterized by the peroxidation of membrane phospholipids due to the generation of ROS [27,28]. The inhibition of ROS by CSE may contribute to ferroptosis resistance in neutrophils, thereby sustaining their continuous activation and promoting the release of NETs. Glutathione peroxidase 4 (GPX4) is considered a key protein in the negative regulation of ferroptosis and is capable of reducing lipid hydroperoxides, preventing ROS accumulation and protecting neutrophils from ferroptosis [29,30]. We hypothesize that CS exposure sustains the release of vital NETosis by inducing neutrophil ferroptosis resistance. Additionally, targeting GPX4-mediated neutrophil ferroptosis resistance may help inhibit NET formation and alleviate glucocorticoid resistance.

## Results

### Abundant NETs formed in COPD patients and were associated with glucocorticoid resistance

Clinical samples were collected from 10 healthy controls and 10 COPD patients with severe pulmonary dysfunction. COPD patients meet the inclusion criteria for the GOLD D group, specifically those who experienced more than 2 moderate exacerbations or 1 hospitalization in the past 1 year [1]. COPD patients selected for inclusion did not include individuals with lung cancer, bilateral multilobular bronchiectasis, or severe infections. The patient details are presented in Table S1. The COPD patients were treated with beta-agonists and muscarinic antagonists combined with inhaled corticosteroids (ICSs), specifically 4 mg of inhaled salbutamol, 0.5 mg of ipratropium bromide, and 2 mg of inhaled budesonide 3 times daily for 5 d. The effectiveness of glucocorticoid treatment was subsequently evaluated via the COPD Assessment Test score: patients with a score of less than 10 were included in the glucocorticoid-sensitive (GC-sensitive) group (*n* = 4), whereas those with a score greater than 10 were included in the glucocorticoid-resistant (GC-resistant) group (*n* = 6). All 6 patients in the GC-resistant group were long-term heavy smokers, suggesting that CS, a common causative factor of COPD, might substantially contribute to glucocorticoid resistance.

**Fig. 1.**
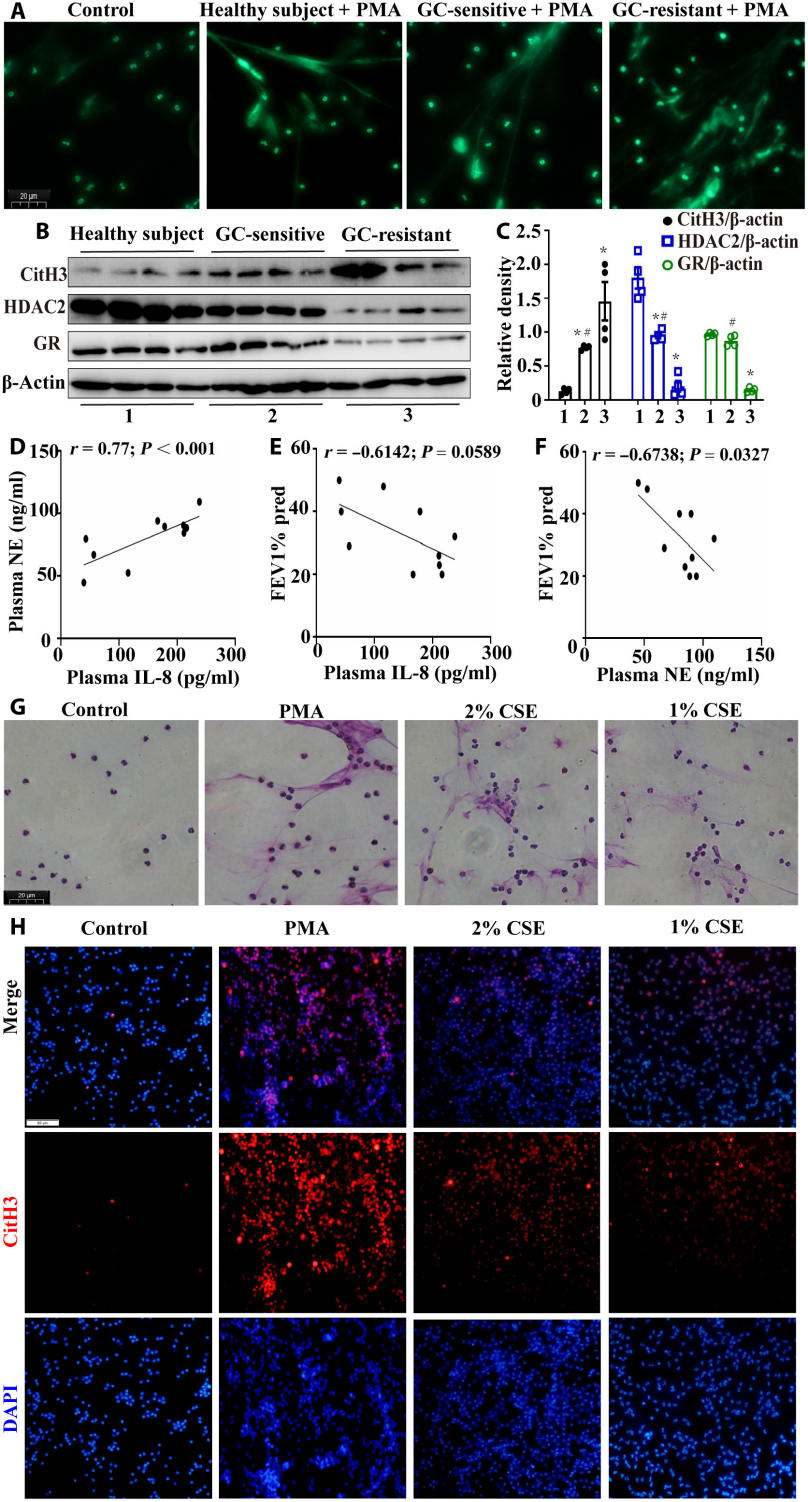
Abundant neutrophil extracellular traps (NETs) formed in chronic obstructive pulmonary disease (COPD) and were associated with glucocorticoid resistance. (A) Neutrophils were stained with SYTOX Green 8 h later (scale bar, 20 μm). (B and C) Western blotting of neutrophils isolated from the peripheral blood of healthy subjects, glucocorticoid-sensitive (GC-sensitive) COPD, and glucocorticoid-resistant (GC-resistant) COPD patients (*n* = 4). (D) Correlation analysis of serum IL-8 and neutrophil elastase (NE) levels in COPD patients was performed (*n* = 10). (E) Correlation analysis of the forced expiration volume in 1 s predicted values (FEV1% pred) and serum IL-8 in COPD patients was conducted (*n* = 10). (F) Correlation analysis of FEV1% pred and serum NE in COPD patients was conducted (*n* = 10). (G) Giemsa staining was performed (scale bar, 20 μm). (H) Results of immunofluorescence staining. Statistical analysis: The data are presented as mean ± SD. Differences were assessed via one-way analysis of variance (ANOVA), followed by Tukey’s post hoc test for significance. Pearson’s correlation tests were conducted for correlation analyses (D to F). *P* < 0.05 indicates a significant difference. *P < 0.05 compared with the control group; ^#^*P* < 0.05 compared with the GC-resistant COPD group. PMA, phorbol 12-myristate 13-acetate; CitH3, citrullinated histone H3; HDAC2, histone deacetylase 2; GR, glucocorticoid receptor; CSE, cigarette smoke extract; DAPI, 4′,6-diamidine-2′-phenylindole dihydrochloride.

Human peripheral blood neutrophils were isolated and divided into 4 groups: the control group (neutrophils from healthy individuals), healthy subject + PMA group (neutrophils from healthy individuals treated with 30 ng/ml PMA), GC-sensitive COPD + PMA group (neutrophils from GC-sensitive COPD patients treated with 30 ng/ml PMA), and GC-resistant COPD + PMA group (neutrophils from GC-resistant COPD patients treated with 30 ng/ml PMA). SYTOX Green staining revealed that NET formation was most pronounced in the GC-resistant group (Fig. 1A). The expression of the NET formation marker citrullinated histone H3 (CitH3) in peripheral blood neutrophils was significantly greater in GC-resistant patients than in healthy subjects and GC-sensitive patients. Moreover, the expression of CitH3 was negatively correlated with the expression of GRα and HDAC2 (Fig. 1B and C). In COPD patients, the expression of IL-8, an indicator of glucocorticoid resistance [31,32], was positively correlated with the expression of NE, a marker associated with NET formation (Fig. 1D). The levels of both markers were negatively correlated with the forced expiration volume in 1 s predicted values (FEV1% pred) (Fig. 1E and F). These findings indicate that NET formation in COPD patients is closely related to glucocorticoid resistance and that glucocorticoid resistance-related NETosis contributes to a decline in lung function. Then, peripheral blood neutrophils from healthy individuals were divided into 4 groups: the control group, the PMA group (stimulated with 30 ng/ml PMA), the 2% CSE group (stimulated with 2% CSE), and the 1% CSE group (stimulated with 1% CSE). We found that CS, a common contributor to COPD development, induced NET formation in peripheral blood neutrophils, similarly to PMA (Fig. 1G and H). Next, we conducted further studies to explore the effect of CS on NET formation and glucocorticoid resistance in COPD through in vivo and in vitro experiments.

### The formation of NETs increased with prolonged exposure to CS in COPD model mice

**Fig. 2.**
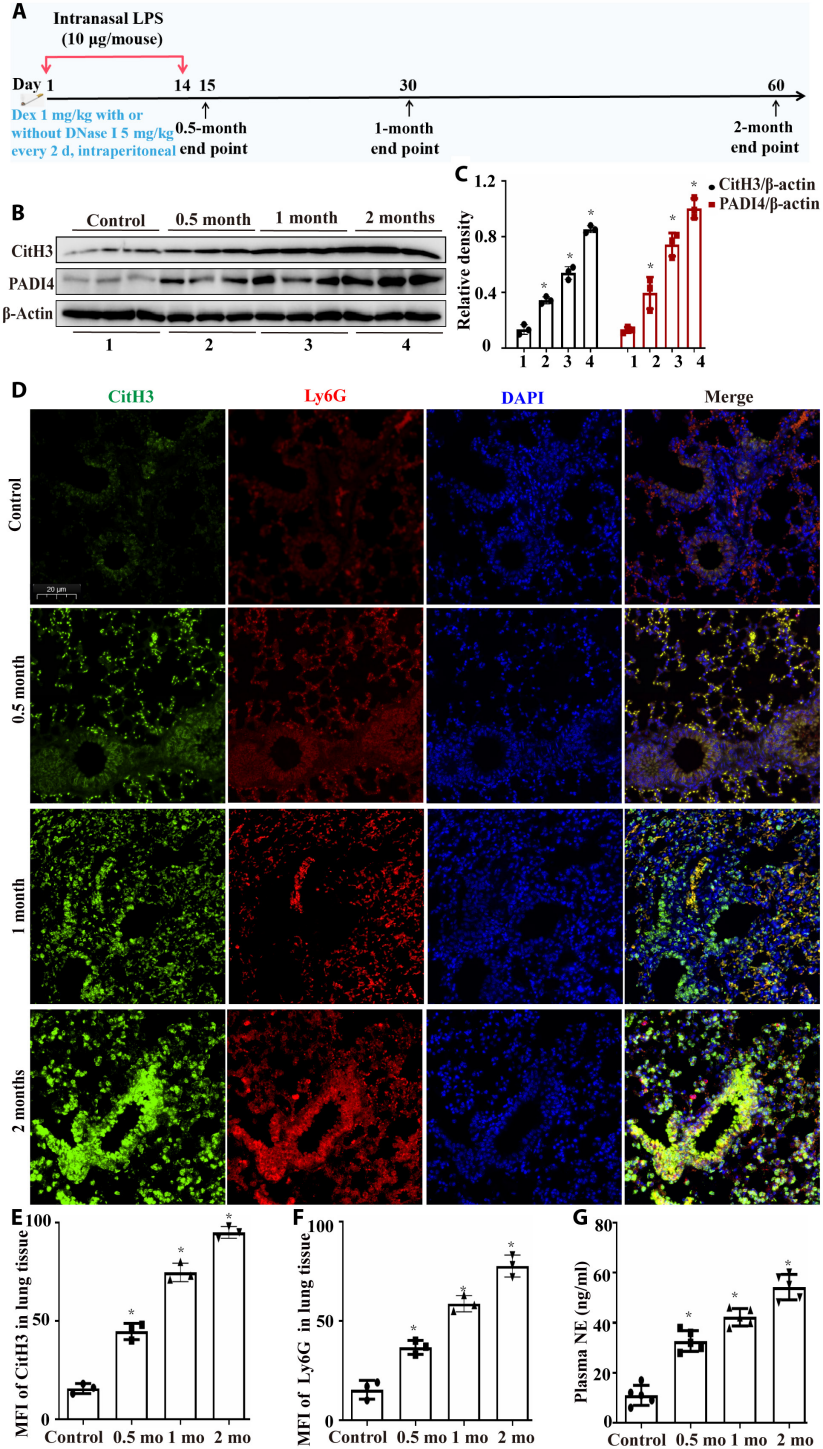
The formation of NETs increased with prolonged exposure to cigarette smoke (CS) in COPD model mice. (A) Schematic diagram of COPD mouse model construction and treatment methods. (B and C) The levels of CitH3 and peptidylarginine deiminase 4 (PADI4) in the lung tissues of mice exposed to CS for different durations were measured using western blotting (*n* = 3). (D) Representative immunofluorescence results demonstrating CitH3 and Ly6G staining in the lung tissues of mice exposed to CS for varying durations (scale bar, 20 μm). (E and F) Mean fluorescence intensity (MFI) of CitH3 and Ly6G in the lung tissues of mice (*n* = 3). (G) Enzyme-linked immunosorbent assay (ELISA) was used to measure serum NE levels in mice exposed to CS for different durations (*n* = 5). Statistical analysis: The data are presented as mean ± SD. Differences were assessed via one-way ANOVA, followed by Tukey’s post hoc test for significance. *P* < 0.05 indicates a significant difference. *P < 0.05 compared with the control group. LPS, lipopolysaccharide; Dex, dexamethasone; DNase I, deoxyribonuclease I.

Western blotting analysis revealed that the expression levels of CitH3 and PADI4 in the lung tissues of mice gradually increased with prolonged CS exposure (Fig. 2B and C). Immunofluorescence staining of lung tissues revealed that the expression of CitH3, an important marker of NET formation, and Ly6G, a marker of neutrophil aggregation, increased with prolonged CS exposure (Fig. 2D to F). The serum level of NE in the mice gradually increased with prolonged CS exposure, as determined by enzyme-linked immunosorbent assay (ELISA) (Fig. 2G). The above results confirmed that the formation of NETs in mice gradually increased with prolonged CS exposure.

### Long-term exposure to CS induced glucocorticoid resistance in a mouse model of COPD

**Fig. 3.**
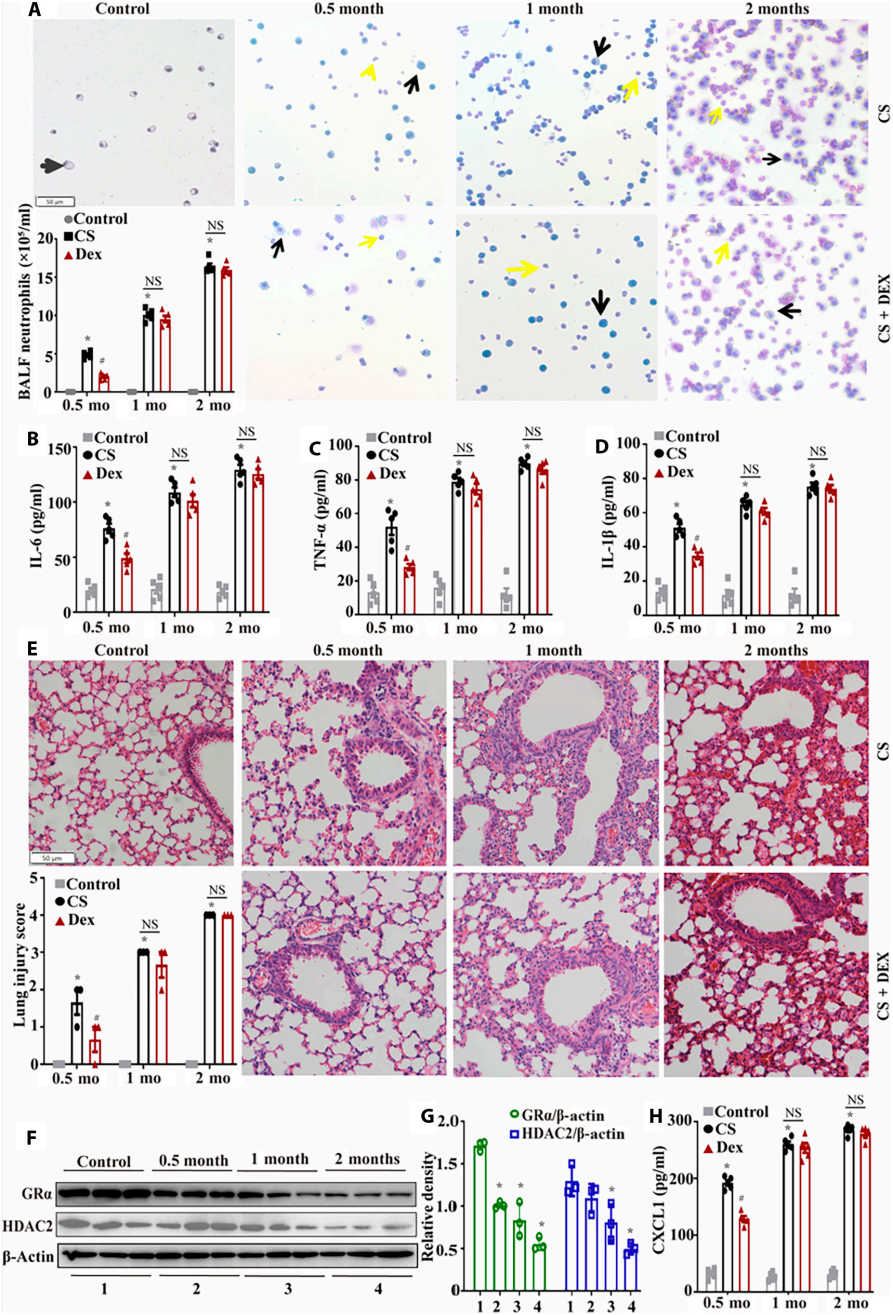
Long-term CS exposure induced glucocorticoid resistance in a mouse model of COPD. Mice were exposed to CS for different durations and treated with or without Dex. (A) Giemsa staining was used to observe inflammatory cell infiltration in bronchoalveolar lavage fluid (BALF; yellow arrow: neutrophils; black arrows: macrophages; scale bar, 50 μm.) (B to D) ELISA was used to measure the levels of inflammatory factors in BALF (*n* = 5). (E) Hematoxylin and eosin (HE) staining was used to assess lung tissue damage (scale bar, 50 μm; *n* = 3). (F and G) Western blotting was used to measure HDAC2 and glucocorticoid receptor α (GRα) levels in mouse lung tissues (*n* = 3). (H) ELISA was performed to measure CXCL1 levels to assess glucocorticoid resistance in mice (*n* = 5). Statistical analysis: The data are presented as mean ± SD. Differences were assessed via one-way ANOVA, followed by Tukey’s post hoc test for significance. *P* < 0.05 indicates a significant difference. *P < 0.05 compared with the control group; ^#^*P* < 0.05 compared with the CS model group; NS, not significant.

Mice were exposed to CS for various durations and treated with or without Dex. We found that with prolonged exposure to CS, there was an increase in inflammatory cell infiltration and the release of inflammatory factors, leading to aggravated lung tissue damage. Dex treatment alleviated lung damage caused by short-term CS exposure (0.5 months) but failed to mitigate lung damage caused by long-term CS exposure (2 months) (Fig. 3A to E). The expression of GRα and HDAC2 decreased with prolonged CS exposure (Fig. 3F and G). In contrast, the expression of CXCL1, a homologous gene of IL-8 in mice, increased with prolonged CS exposure (Fig. 3H). These results suggested that prolonged CS exposure induced glucocorticoid resistance in COPD model mice.

### Targeting CS-induced NETs rescued glucocorticoid resistance in COPD

**Fig. 4.**
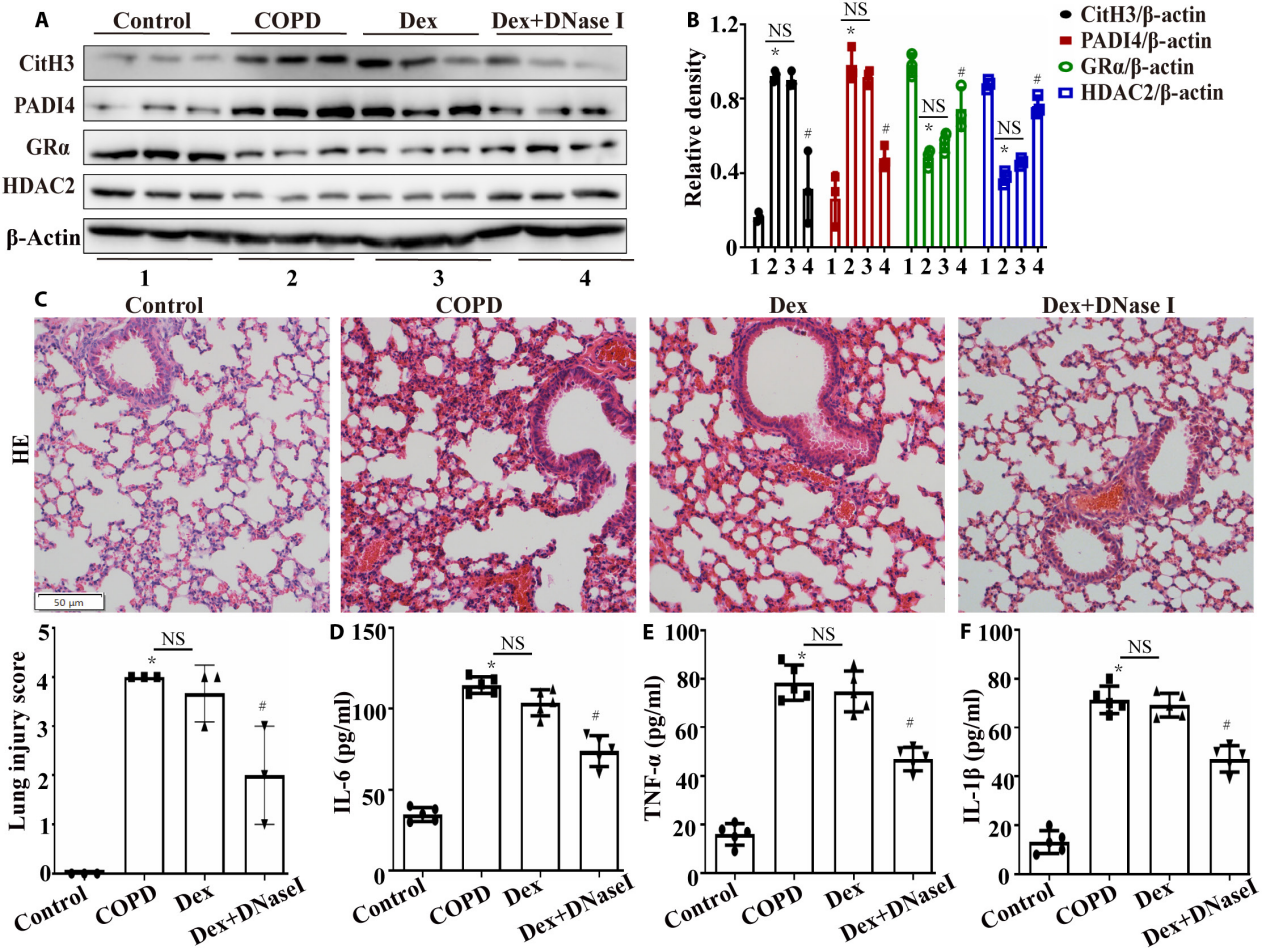
NETs played a vital role in glucocorticoid resistance in COPD. (A and B) Western blotting was used to measure NET formation and the levels of glucocorticoid-sensitivity-related proteins in mouse lung tissues (*n* = 3). (C) HE staining was utilized to evaluate lung injury in mice (scale bar, 50 μm; *n* = 3). (D to F) Inflammatory factor levels in BALF were measured by ELISA (*n* = 5). Statistical analysis: The data are presented as mean ± SD. Differences were assessed via one-way ANOVA, followed by Tukey’s post hoc test for significance. *P* < 0.05 indicates a significant difference. *P < 0.05 compared with the control group; ^#^*P* < 0.05 compared with the COPD model group; NS, not significant. Dex+DNase I, COPD + Dex + 5 mg/kg DNase I every 2 d, intraperitoneal.

To investigate NETs’ role in glucocorticoid resistance, we established 4 experimental groups: the control group, the COPD group (exposed to CS for 2 months), the Dex group (COPD + 1 mg/kg Dex every 2 d, intraperitoneal), and the Dex+DNase I group (COPD + Dex + 5 mg/kg deoxyribonuclease I [DNase I] every 2 d, intraperitoneal). Notably, DNase I co-administration with Dex significantly decreased CitH3 and PADI4 expression while up-regulating HDAC2 and GRα compared to Dex monotherapy (Fig. 4A and B), indicating that NET degradation restored glucocorticoid sensitivity in CS-induced COPD. Furthermore, the combination therapy attenuated CS-induced lung pathology and inflammatory responses (Fig. 4C to F).

### Exposure to CSE sustained the activation and release of inflammatory mediators by inducing ferroptosis resistance in neutrophils

**Fig. 5.**
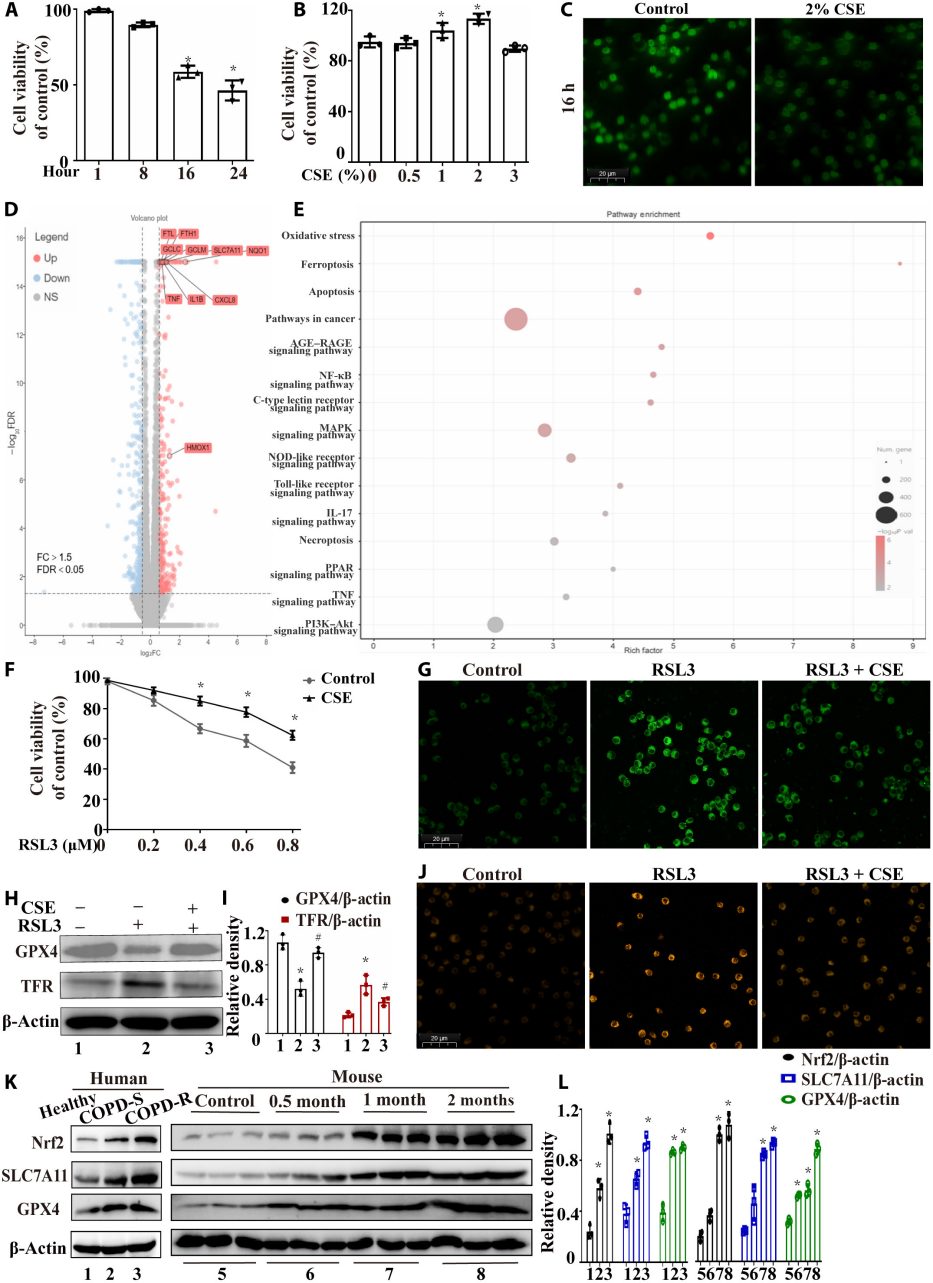
Exposure to CSE sustained neutrophil activation by inducing ferroptosis resistance in vitro. (A and B) Cell viability was evaluated via a Cell Counting Kit-8 (CCK-8) assay (*n* = 3). (C) Neutrophils were divided into 2 groups: the control group and the 2% CSE exposure group. The reactive oxygen species (ROS) levels in the 2 groups were compared after 16-h isolation in vitro (scale bar, 20 μm). (D) Volcano plot of the RNA sequencing (RNA-seq) data. (E) The top 15 pathways in which the up-regulated genes were enriched. (F) Cell viability was measured by a CCK-8 assay (*n* = 3). (G) Lipid peroxide levels were measured via a Liperfluo assay (scale bar, 20 μm). (H and I) Western blotting was used to measure the expression of glutathione peroxidase 4 (GPX4) and transferrin receptor (TFR) in neutrophils (*n* = 3). (J) FerroOrange staining was used to detect Fe^2+^ in neutrophils (scale bar, 20 μm). (K and L) The nuclear factor erythroid 2-related factor 2 (Nrf2)/solute carrier family 7 member 11 (SLC7A11)/GPX4 pathway was detected in neutrophils isolated from the peripheral blood of humans and mice. Statistical analysis: The data are presented as mean ± SD. Differences were assessed via one-way ANOVA, followed by Tukey’s post hoc test for significance. *P* < 0.05 indicates a significant difference. *P < 0.05 compared with the control group; ^#^*P* < 0.05 compared with the RSL3 model group. FDR, false discovery rate; FC, fold change; COPD-S, COPD GC-sensitive; COPD-R, COPD GC-resistant.

Neutrophils are terminally differentiated cells lacking proliferative capacity, with their viability gradually declining after isolation. A significant decrease was observed at 16 h, exhibiting a half-life of approximately 24 h (Fig. 5A and Fig. S2). Neutrophil viability was detected after 8-h stimulation with various CSE concentrations (Fig. 5B). Compared to the control group, CSE exposure enhanced neutrophil survival, peaking at 2% CSE. This finding presents a paradox: CSE is extensively documented to reduce the viability of Beas-2B cells (a bronchial epithelial cell line central to COPD research), and our prior study confirmed 2% CSE as a relatively high cytotoxic dose that could cause significant damage to Beas-2B cells [33]. Interestingly, 2% CSE improved neutrophil viability in this study. However, higher concentrations (e.g., 3% CSE) not only failed to enhance viability but caused a slight reduction versus the control group, possibly attributable to the toxic components (e.g., heavy metals, nitrosamines, and aldehydes) in CSE exerting cellular damage at excessive doses. We also found that 2% CSE exposure inhibited ROS production in neutrophils (Fig. 5C). Furthermore, as neutrophil activity increased, the release of inflammatory factors and NETs also increased (Fig. S3). We subsequently performed RNA sequencing analysis to explore the mechanism through which CSE inhibits neutrophil death. A total of 16,719 genes were analyzed, and we found that 280 genes were up-regulated and 310 genes were down-regulated following CSE exposure. The results from the transcriptome sequencing, violin plot, sample correlation, and sample principal component analysis indicated significant differences among the groups of sequencing data, with good reproducibility (Fig. S4). The volcano plot revealed a significant up-regulation of inflammation-related and ferroptosis-related genes following CSE exposure (Fig. 5D). Figure 5E shows the top 15 pathways in which the up-regulated genes were enriched, and the results suggested that CSE promoted the up-regulation of ferroptosis-related genes in neutrophils. We hypothesize that exposure to CSE induced neutrophils to develop resistance to ferroptosis, thereby sustaining their persistent activation and promoting the release of inflammatory mediators. We subsequently conducted further in vitro validation experiments using the ferroptosis inducer RSL3.

To further elucidate the effect of CSE on neutrophil ferroptosis, we stimulated neutrophils with the ferroptosis inducer RSL3. As the concentration of RSL3 gradually increased, the viability of neutrophils gradually decreased. At a concentration of 0.8 μM RSL3, neutrophil activity decreased to approximately 50%. However, neutrophils treated with 2% CSE showed a relatively high viability in response to RSL3 stimulation compared to the control group (Fig. 5F). Then, neutrophils were divided into 3 groups: the control group, 0.4 μM RSL3 group, and 0.4 μM RSL3 + 2% CSE treatment group. Eight hours later, the cells were collected, and the lipid peroxide levels were measured via a Liperfluo assay. We found that CSE exposure reversed the increase in lipid peroxidation levels induced by RSL3 in neutrophils (Fig. 5G). The western blotting results demonstrated that CSE exposure increased GPX4 expression and decreased transferrin receptor (TFR) expression in neutrophils following RSL3 stimulation (Fig. 5H and I). FerroOrange staining revealed that CSE exposure reversed the elevation of intracellular Fe^2+^ levels induced by RSL3 (Fig. 5J). Subsequently, we isolated neutrophils from the peripheral blood of healthy, COPD GC-sensitive, and COPD GC-resistant individuals and mice with different durations of CS exposure and analyzed ferroptosis-related proteins using western blot. We found that compared to that in the healthy control group, the nuclear factor erythroid 2-related factor 2 (Nrf2)/solute carrier family 7 member 11 (SLC7A11)/GPX4 pathway was significantly up-regulated in COPD patients, with the most notable increase observed in the COPD GC-resistant group. In the COPD model mice, Nrf2, SLC7A11, and GPX4 gradually increased with prolonged exposure to CS (Fig. 5K and L). These results suggested that CS exposure induced ferroptosis resistance in neutrophils.

### RSL3 inhibited NET formation and increased glucocorticoid sensitivity in vitro

**Fig. 6.**
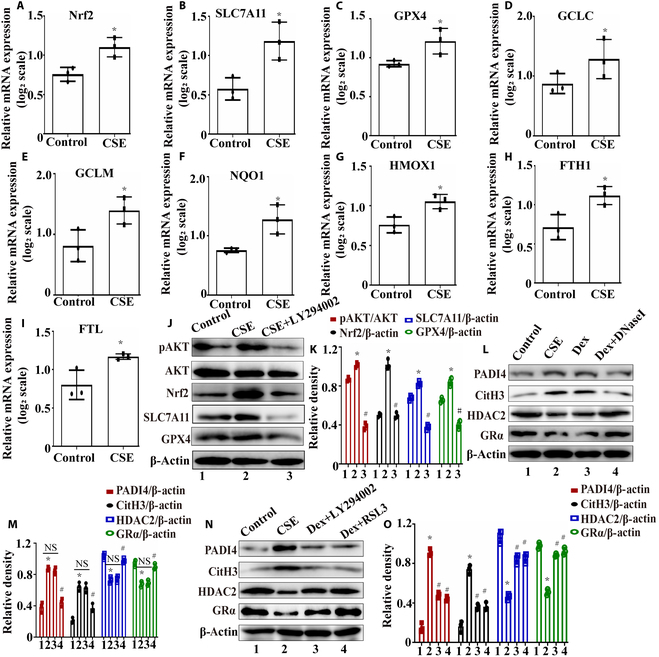
RSL3 inhibited NET formation and improved glucocorticoid resistance in vitro. (A to I) The effect of 2% CSE exposure on the expression of genes related to ferroptosis in neutrophils was assessed by quantitative real-time polymerase chain reaction (qPCR). (J and K) Westen blotting results of phosphorylation AKT and the Nrf2/SLC7A11/GPX4 pathway. (L and M) Neutrophils isolated from the peripheral blood of healthy individuals were divided into 4 groups: the control group, 2% CSE exposure group, Dex group (2% CSE + 1 μM Dex), and Dex+DNase I group (2% CSE + 1 μM Dex + 0.1 mg/ml DNase I). After 8 h of treatment, the cells were collected, and the expression levels of PADI4, CitH3, HDAC2, and GRα were compared using western blotting. (N and O) Neutrophils were divided into 4 groups: the control group, 2% CSE exposure group, Dex+LY294002 group (2% CSE + 1 μM Dex + 10 μM LY294002), and Dex+RSL3 group (2% CSE + 1 μM Dex + 0.4 μM RSL3). After 8 h of treatment, the cells were collected, and the expression levels of peptidylarginine deiminase 4 (PADI4), CitH3, HDAC2, and GRα were compared using western blotting. Statistical analysis: The data are presented as mean ± SD. Differences were assessed via one-way ANOVA, followed by Tukey’s post hoc test for significance. *P* < 0.05 indicates a significant difference. *P < 0.05 compared with the control group; ^#^*P* < 0.05 compared with the CSE model group; NS, not significant. mRNA, messenger RNA.

Pathway enrichment analysis indicated that CSE significantly up-regulated genes associated with ferroptosis in neutrophils. Then, we performed quantitative real-time polymerase chain reaction (qPCR) analysis on the relevant genes (Fig. 6A to I). Our previous research has confirmed that the Nrf2/SLC7A11/GPX4 pathway plays a crucial role in activating antioxidant stress genes (such as HMOX1 and NQO1) and inhibiting ferroptosis [34,35]. The qPCR results in this study revealed that CSE exposure activated the Nrf2/SLC7A11/GPX4 pathway in neutrophils and promoted the expression of downstream antioxidant factors, including GCLC, GCLM, NQO1, and HMOX1 (Fig. 6A to G). Hosseinzadeh et al. [13] reported that nicotine-induced NET formation depends on AKT and PADI4 activation. We hypothesize that CSE mediates neutrophil ferroptosis resistance by promoting AKT phosphorylation and activating the Nrf2/SLC7A11/GPX4 pathway. We confirmed this hypothesis through western blotting (Fig. 6J and K). Neutrophils from the peripheral blood of healthy individuals were divided into 3 groups: the control group, the 2% CSE exposure group, and the 2% CSE + 10 μM LY294002 group. After 8 h of treatment, the cells were collected for western blotting. We found that simultaneous treatment of neutrophils with CSE and LY294002 inhibited AKT phosphorylation and down-regulated the expression of Nrf2, SLC7A11, and GPX4 (Fig. 6J and K). Additionally, we found that CSE promoted the expression of FTH1 and FTL in neutrophils (Fig 6H and I). Recent studies have found that ferritin, composed of FTH1 and FTL, plays a significant role in activating neutrophils to generate NETs by promoting PADI4 [36,37]. In summary, CSE stimulated the expression of genes related to antioxidant stress in neutrophils, thereby maintaining their activity. Additionally, it promoted the release of ferritin, which induced an increase in PADI4 expression. Ultimately, CSE promoted the formation of “vital” NETs in neutrophils through resistance to ferroptosis.

We further investigated the relationships among CSE-induced ferroptosis resistance, NET formation, and glucocorticoid resistance in neutrophils. Although there was research [38] about Dex (1 μM) being able to inhibit the expression of PADI4 in inflammatory cells, we found that CSE stimulation decreased the sensitivity of neutrophils to Dex in vitro. After NETs had been eliminated using DNase I, the sensitivity of neutrophils to Dex was restored in vitro (Fig. 6L and M). Furthermore, we found that the induction of ferroptosis by an AKT phosphorylation inhibitor or a GPX4 inhibitor suppressed CSE-induced NET formation and increased glucocorticoid sensitivity in neutrophils (Fig. 6N and O). That is to say, inducing ferroptosis in neutrophils can inhibit the formation of NETs and increase glucocorticoid sensitivity.

### Neutrophil-specific GPX4 knockout alleviated neutrophil infiltration and suppressed the formation of NETs in COPD model mice

**Fig. 7.**
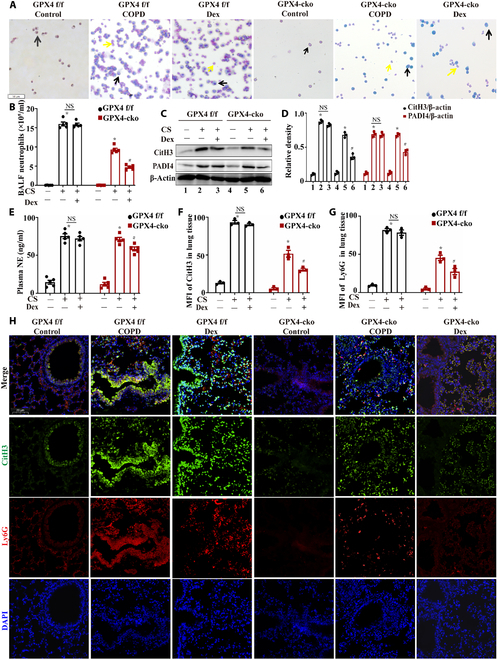
Neutrophil-specific GPX4 knockout alleviated neutrophil infiltration and suppressed the formation of NETs in COPD mice. (A) Giemsa staining results of BALF obtained from GPX4 f/f and GPX4-cko mice (yellow arrow: neutrophils; black arrows: macrophages. scale bar, 50 μm.). (B) Neutrophil counts in the BALF of GPX4 f/f and GPX4-cko mice (*n* = 5). (C and D) Western blotting of PADI4 and CitH3 expression in the lung tissues of GPX4 f/f and GPX4-cko mice (*n* = 3). (E) Measurement of serum NE levels in GPX4 f/f and GPX4-cko mice by ELISA (*n* = 5). (F and G) MFIs of CitH3 and Ly6G in the lung tissues of mice (*n* = 3). (H) Representative immunofluorescence results for CitH3 and Ly6G in the lung tissues of GPX4 f/f and GPX4-cko mice (scale bar, 20 μm). Statistical analysis: The data are presented as mean ± SD. Differences were assessed via one-way ANOVA, followed by Tukey’s post hoc test for significance. *P* < 0.05 indicates a significant difference. *P < 0.05 compared with the control group; ^#^*P* < 0.05 compared with the COPD model group; NS, not significant.

We subsequently used neutrophil-specific GPX4 knockout (GPX4-cko) mice to investigate the effects of prolonged CS exposure on NET formation after the correction of neutrophil ferroptosis resistance in COPD. Both GPX4 f/f and GPX4-cko mice were divided into 3 experimental groups: the control group, COPD group (exposed to CS for 2 months), and Dex group (exposed to CS for 2 months and administered Dex at a dose of 1 mg/kg every 2 d by intraperitoneal injection). Upon prolonged CS exposure, the number of inflammatory cells in the bronchoalveolar lavage fluid (BALF) was reduced in GPX4-cko mice compared to that in GPX4 f/f mice, and Dex treatment significantly decreased neutrophil infiltration in GPX4-cko mice (Fig. 7A and B). Compared with GPX4 f/f mice, GPX4-cko COPD mice exhibited lower NET formation in lung tissues. Furthermore, Dex treatment markedly inhibited NET formation in GPX4-cko COPD model mice, whereas it had a weak effect on NET formation in GPX4 f/f COPD mice (Fig. 7C to H). Specifically, following Dex treatment, the expression levels of PADI4 and CitH3 in the lung tissues of GPX4-cko COPD mice decreased (Fig. 7C and D), serum NE levels declined (Fig. 7E), and immunofluorescence analysis revealed reduced expression levels of CitH3 and Ly6G in lung tissues (Fig. 7F to H).

### Neutrophil-specific GPX4 knockout increased glucocorticoid sensitivity in COPD

**Fig. 8.**
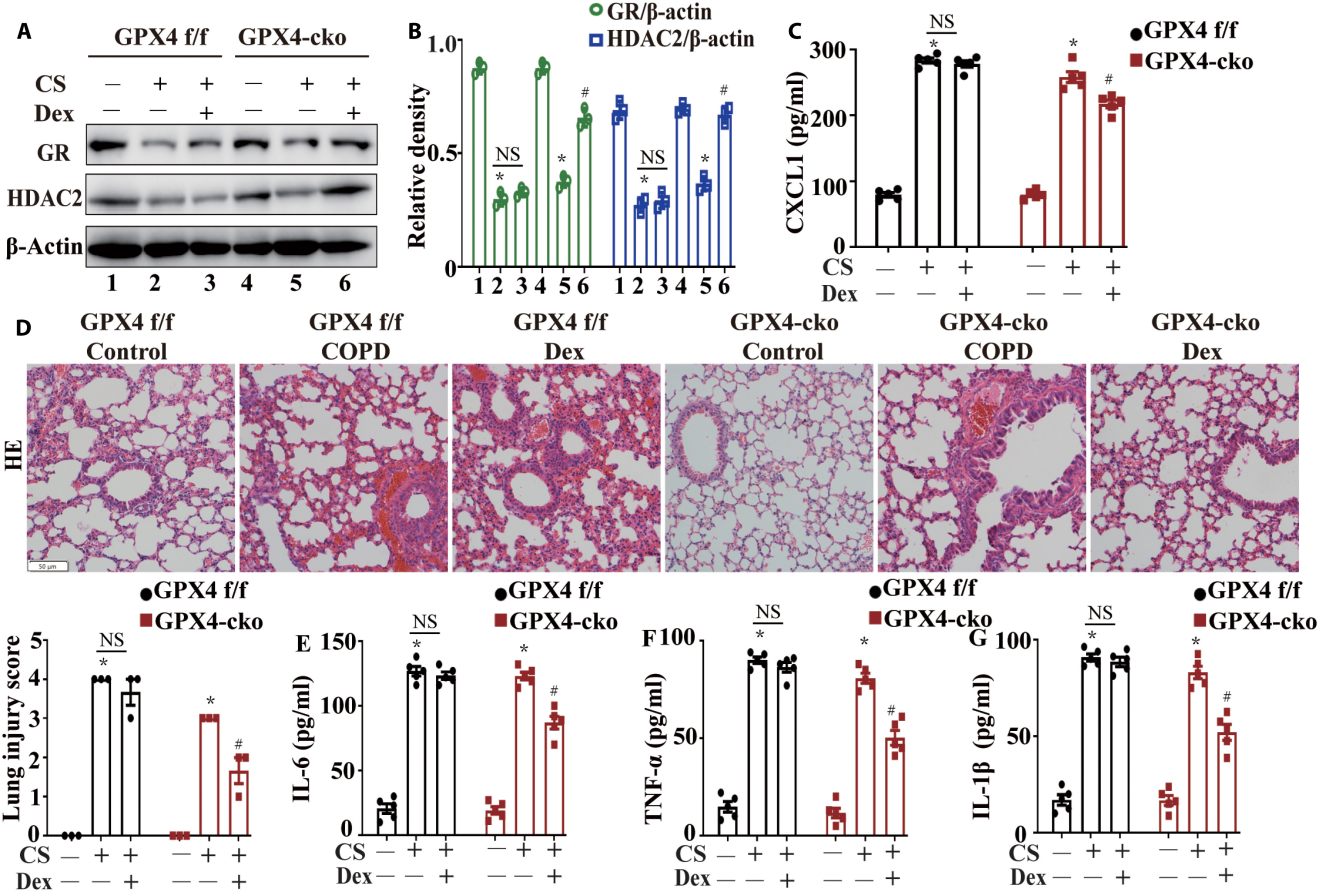
Neutrophil-specific GPX4 knockout increased glucocorticoid sensitivity in COPD. (A and B) Western blotting of GRα and HDAC2 expression in the lung tissues of GPX4 f/f and GPX4-cko mice (*n* = 3). (C) Measurement of serum CXCL1 levels in GPX4 f/f and GPX4-cko mice by ELISA (*n* = 5). (D) Representative images of HE staining results of lung tissues from GPX4 f/f and GPX4-cko mice (scale bar, 50 μm; *n* = 3). (E to G) Measurement of inflammatory markers in the BALF of GPX4 f/f and GPX4-cko mice by ELISA (*n* = 5). Statistical analysis: The data are presented as mean ± SD. Differences were assessed via one-way ANOVA, followed by Tukey’s post hoc test for significance. *P* < 0.05 indicates a significant difference. *P < 0.05 compared with the control group; ^#^*P* < 0.05 compared with the COPD model group; NS, not significant.

After confirming that neutrophil-specific knockout of GPX4 inhibited the formation of NETs in COPD mice, we further investigated its effect on the glucocorticoid sensitivity in COPD. Following Dex treatment, the GRα and HDAC2 levels in GPX4-cko COPD model mice were significantly elevated, whereas serum CXCL1 levels decreased markedly; in contrast, in GPX4 f/f COPD mice, the levels of these proteins were not significantly normalized after Dex treatment (Fig. 8A to C). Neutrophil-specific knockout of GPX4 increased glucocorticoid sensitivity in COPD model mice, as demonstrated by a significant reduction in lung damage in GPX4-cko COPD model mice (Fig. 8D) and a marked decrease in the levels of inflammatory factors in the BALF after Dex treatment (Fig. 8E to G).

## Discussion and Conclusion

COPD is a chronic inflammatory airway condition primarily characterized by irreversible airflow obstruction. Although bronchodilators remain the first-line treatment for COPD, ICSs have become an important adjunct therapy for patients with severe lung function impairment because of their significant ability to suppress airway inflammation, reduce the frequency of acute exacerbations, and decrease mortality risk [1,39]. According to the 2024 GOLD guidelines the use of the ICSs is recommended for GOLD group C or D patients with eosinophil (EOS) counts ≥100 cells/μl [1]. In clinical practice, most COPD patients with an EOS count ≥300 cells/μl indeed respond well to ICSs; however, some COPD patients with a history of long-term smoking and an EOS count between 100 and 300 cells/μl do not benefit from ICS treatment. Some heavy smokers demonstrate a poor response to glucocorticoids. Our study indicates that NET formation induced by CS significantly contributes to glucocorticoid resistance. We found that the expression of NE, an indicator of NET formation, was positively correlated with IL-8 levels and negatively correlated with the FEV1%. Thus, we propose that NE levels could serve as a valuable reference indicator for glucocorticoid resistance in patients with COPD.

NET formation in the body acts as a “double-edged sword”: it can provide protection by reducing external pathogen stimulation, but it may also amplify inflammation and cause tissue damage [19,40]. Intracellular and extracellular microbes, as well as physical and chemical stimuli, induce the formation of NETs, which prevent microbial spread to secondary sites and exert direct antibacterial effects [19]. However, excessive or inefficient degradation of NETs can harm the host, leading to uncontrolled inflammatory responses and immune dysregulation [40]. Excessive formation of NETs has been shown to play an important role in glucocorticoid resistance in asthma [17,32]. Additionally, our study confirms that NETs also contribute to glucocorticoid resistance in COPD. Studies have shown that CS damages airway epithelial cells, leading to extensive recruitment of neutrophils that secrete various serine proteases and inflammatory mediators, including NE and MPO, which promote NET formation [12,41]. NETs can cause the recruitment of additional monocytes/macrophages and T helper 17 cells, establishing a positive feedback loop that stimulates the release of substantial amounts of inflammatory mediators, including TNF-α, IL-17, IL-1β, IL-6, and IL-8 [41–43]. TNF-α inhibits GRα expression [44]; IL-17 promotes GRβ up-regulation and suppresses HDAC2 expression [6,45]; IL-1β activates the inflammatory pathway by up-regulating TLR2, which works in conjunction with glucocorticoid resistance [46]; IL-6 down-regulates the expression of GRα by disrupting the negative feedback regulation of the hypothalamic–pituitary–adrenal axis [47]; and IL-8 can induce glucocorticoid resistance by influencing the phosphorylation of GRα at Ser211 [31]. In summary, neutrophil infiltration, NET formation, and the release of inflammatory factors reinforce each other, ultimately resulting in glucocorticoid resistance, which is consistent with our research findings. In our COPD animal model, long-term CS exposure caused increased neutrophil aggregation and NET formation, which caused a decrease in HDAC2 and an increase in IL-8 levels. Meanwhile, the decrease in HDAC2 further recruited more neutrophils, enhanced the release of NETs, and led to a further reduction in HDAC2, perpetuating a vicious cycle. Following the removal of NETs via DNase I, the sensitivity of COPD mice to Dex was restored, as indicated by reduced airway inflammation and alleviated lung tissue damage. These findings suggest that the formation of NETs plays an important role in glucocorticoid resistance in COPD.

Consistent with previous research findings [13], we found that CSE promoted vital NETosis by stimulating PADI4 expression, independent of ROS. As noted by Ng et al.[25], neutrophils are short-lived cells. In vitro, we observed that the activity of neutrophils began to decline 16 h after isolation and decreased by 50% after 24 h. How are the long term-recruitment of short-lived neutrophils to target organs and the release of substantial amounts of vital NETs achieved? At present, no definitive conclusion has been reached. Yang et al. [48] suggested that external stimuli induce the transformation of neutrophils into “aged” neutrophils, thereby prolonging their survival and maintaining their activity. Hosseinzadeh et al. [13] suggested that external stimuli promote AKT phosphorylation in neutrophils, thereby inhibiting apoptosis. In this study, we performed single-cell RNA sequencing and discovered that CSE induced neutrophil ferroptosis resistance. We found that CSE not only up-regulated the expression of inflammatory genes such as TNF-α, IL-1β, and IL-8 but also increased the expression of genes related to antioxidant stress and iron storage, specifically FTH1 and FTL, in neutrophils. Wang et al. [49] reported that the up-regulation of FTH1 in neutrophils can reduce their susceptibility to oxidative stress, increase their proinflammatory effects, and prolong their residence time in the lungs. Furthermore, recent research has shown that ferritin, composed of FTH1 and FTL, plays an important role in the release of inflammatory factors and the formation of NETs [37,50]. This result is consistent with our findings that resistance to ferroptosis in neutrophils, resulting in increased ferritin, can induce inflammatory responses and NET formation. On the basis of these findings, we believe that CSE induces neutrophil ferroptosis resistance, which helps maintain cell viability and promotes ferritin synthesis. Together, these effects facilitate the continuous formation of “vital” NETs, mediating inflammatory responses that lead to lung tissue damage and glucocorticoid resistance.

The Nrf2/SLC7A11/GPX4 pathway is the most well-known classic pathway for resisting ferroptosis and can be activated by AKT phosphorylation [27,51]. In our study, we confirmed the results of pathway enrichment analysis showing that CSE activates the Nrf2/SLC7A11/GPX4 pathway by promoting neutrophil AKT phosphorylation through qPCR and western blotting. Research has shown that following AKT phosphorylation, Nrf2 is released from Keap1, which enhances its activity. This, in turn, facilitates the entry of Nrf2 into the nucleus and regulates the expression of the downstream key ferroptosis core genes SLC7A11 and GPX4 [51,52]. SLC7A11 transports cystine into the cells, where it serves as a precursor for the production of the antioxidant glutathione. GPX4 subsequently utilizes glutathione to neutralize lipid ROS, thereby preventing ferroptosis [53]. For inflammatory cells, resistance to ferroptosis has the same effect as enhanced proinflammatory effects and exacerbates the detrimental impact of inflammation on the body. In this study, we found that increased GPX4 transcription, inhibition of iron-dependent lipid peroxidation, restoration of cellular redox homeostasis, and reduced cell sensitivity to ferroptosis led to sustained neutrophil activation and promoted NET formation, which affected the glucocorticoid response. We subsequently used RSL3 in vitro and neutrophil-specific GPX4-cko mice in vivo to confirm that inhibiting neutrophil GPX4 expression can suppress NET formation, thereby increasing glucocorticoid sensitivity in COPD.

We must acknowledge that although Liperfluo staining can detect lipid peroxides, it may also be influenced by other strong oxidants, such as hypochlorous acid produced by MPO in neutrophils and peroxynitrite. Therefore, the lack of specificity of Liperfluo staining for lipid peroxidation must be acknowledged.

In conclusion, CS induces neutrophil ferroptosis resistance by promoting AKT phosphorylation and activating the Nrf2/SLC7A11/GPX4 pathway, which sustains neutrophil activation, increases NET release and mediates the down-regulation of GRα and HDAC2, ultimately driving glucocorticoid resistance in COPD.

## Materials and Methods

### Materials

The cigarette used in this study was Hongtashan. Lipopolysaccharide (LPS), DNase I), PMA, LY294002 (specific inhibitors of pAKT), and RSL3 were purchased from Sigma (St. Louis, MO, USA). Dex was purchased from Macklin (Shanghai, China). Cell Counting Kit-8 was from Life-iLab (Shanghai, China). Cell culture reagents were purchased from Zhongqiaoxinzhou Biotech (Shanghai, China). The Liperfluo probe was provided by Dojindo (Kyushu, Japan). The 2′,7′-dichlorodihydrofluorescein diacetate (DCFH-DA) fluorescent probe and calcein/propidium iodide (PI) cell viability/cytotoxicity assay kit were from Beyotime Biotechnology (Shanghai, China). The SYTOX staining kit was from X-Y Biotechnology (Shanghai, China). Human and mouse peripheral blood neutrophil isolation kits were from Solarbio (Beijing, China). Antibodies, including anti-Nrf2), anti-PADI4, anti-HDAC2, anti-β-actin, anti-GPX4, and anti-TFR antibodies, were from Zenbio (Chengdu, China). Anti-CitH3 and anti-SLC7A11 were provided by Abcam (Cambridge MA, USA). Anti-GRα and anti-HDAC2 were from ABclonal (Wuhan, China). Anti-phospho-AKT antibodies and anti-AKT were provided by Cell Signaling Technology (Danvers, USA). The qPCR primers were synthesized by Comatebio (Jilin, China).

### Isolation and processing of primary neutrophils

Following approval from the Ethics Committee of the First Hospital of Jilin University, human (consent was obtained) and mouse peripheral blood were collected, and primary neutrophils were isolated using peripheral blood neutrophil isolation kits. The purity of the extracted neutrophils was confirmed to be greater than 95% via Giemsa staining. Neutrophils isolated from peripheral blood were resuspended in RPMI 1640 medium supplemented with 10% fetal bovine serum and incubated at 37 °C with 5% CO_2_.

### Animal experiments

The animal studies received approval from the First Hospital of Jilin University’s Ethics Committee. Wild-type C57BL/6 mice (6 to 8 weeks old; weight: 16 to 20 g) were provided by Qianhe Technology Industrial (Jilin, China). The COPD model was established by combining CS exposure with intranasal LPS administration based on our previous methods [33]: The PAB-S200 animal passive smoking exposure system (Biolab Technology, Beijing, China) was used to expose mice to CS. Three cigarettes were lit each session, exposure lasting 1 h, followed by a 2-h interval. This process was repeated 4 times daily, totaling 12 cigarettes per day. Additionally, on the 1st and 14th days of the experiment, LPS (10 μg/mouse) nasal drops were administered to the mice to enhance COPD model formation. Based on the duration of CS exposure and the administration of Dex treatment, the mice were divided into 8 groups, with 10 mice per group: the control group, the 0.5-month CS group (CS exposure for 0.5 months), the 0.5-month Dex group (CS exposure for 0.5 months and treated with Dex at a dose of 1 mg/kg every 2 d by intraperitoneal injection), the-1 month CS group (CS exposure for 1 month), the 1-month Dex group (CS exposure for 1 month and treated with Dex), the 2-month CS group (CS exposure for 2 months), the 2-month Dex group (CS exposure for 2 months and treated with Dex), and the Dex+DNase I group (CS exposure for 2 months and treated with both Dex and DNase I at a dose of 5 mg/kg every 2 d by intraperitoneal injection). The schematic diagram of COPD mouse model construction and treatment methods is shown in Fig. 2A.

### Histological analysis

Mouse lung tissues from distinct groups were fixed in 4% paraformaldehyde for 48 h, after which the tissues were dehydrated and embedded in paraffin. The tissue sections (4 to 5 μm) were routinely stained with hematoxylin and eosin. The lung injury score was assessed using a semiquantitative scoring method as previously described [34]: Lung tissue damage was evaluated on a scale of 0 to 4 based on the percentage of injury: 0% to 10% (score 0), 11% to 25% (score 1), 26% to 50% (score 2), 51% to 75% (score 3), and 76% to 100% (score 4).

### Immunofluorescence staining of lung tissues

Paraffin sections (3 μm) from mouse lungs were deparaffinized in xylene. Antigen retrieval was subsequently performed by 6-min pressure cooking in citrate buffer, followed by a 2.5% bovine serum albumin blocking step. Sections were incubated with anti-CitH3 and anti-Ly6G primary antibodies overnight at 4 °C. Following 3 washing steps, diluted biotinylated secondary antibodies were incubated at room temperature for 30 min. Subsequently, 4′,6-diamidine-2′-phenylindole dihydrochloride (DAPI) was used for nuclear staining. The normalized mean fluorescence intensity areas in lung sections were calculated using ImageJ.

### Analysis of BALF

The BALF samples were obtained via endotracheal instillation of sterile phosphate-buffered saline (PBS). Following collection, the BALF was spun at 5,000 rpm for 5 min under 4 °C conditions. The resulting supernatant was utilized for assessing inflammatory cytokines through ELISA. Meanwhile, the cell pellets were reconstituted in 200 μl of RPMI 1640 medium. Referring to previous studies [17], the total cell number in BALF was counted using a hemocytometer, while the number of neutrophils was determined in a total of 200 BALF cells after Wright–Giemsa staining.

### Wright–Giemsa staining

The Giemsa staining method for mouse BALF was as follows: After mouse BALF was resuspended, 50 μl of RPMI 1640 was placed in the sample wells of a cytocentrifuge, and the cells were plated on slides via centrifugation. After methanol fixation, Giemsa staining solutions A and B (Baso, Wuhan, China) were sequentially added at a 1:3 ratio by volume. After 5 min, the staining solution was rinsed away with running water, and the samples were observed under a microscope.

The steps for Giemsa staining of blood primary neutrophils were as follows: Neutrophils were first isolated from human and mouse peripheral blood and then plated at a density of 2 × 10^6^ cells per well onto poly-l-lysine-coated coverslips in a 24-well plate. After experimental treatments, the cells were subjected to Giemsa staining following an 8-h incubation period.

### Detection of inflammatory markers

The levels of human IL-8 (MLbio, Shanghai, China), human NE (MLbio, Shanghai, China), mouse CXCL1 (MLbio, Shanghai, China), mouse TNF-α, mouse IL-6, and mouse IL-1β (BioLegend, CA, USA) in serum, BALF, or the culture medium of cells were analyzed by commercial ELISA kits.

### Identification and breeding of mice with neutrophil-specific GPX4 deletion

The Cre/loxP system was used to generate S100a8-Cre^+^GPX4^flox/flox^ mice, referred to as GPX4-cko mice, enabling lineage-specific deletion of GPX4 exclusively in neutrophils [54]. C57BL/6J-GPX4em1Cflox/Cya (referred to as GPX4 f/f) and S100a8-Cre-EGFP mice were purchased from Cyagen (Jiangsu, China). Tail samples were collected, and DNA was extracted via the Mouse Direct PCR Kit (Selleck, Houston, USA). Polymerase chain reaction (PCR) was then performed using the following primers for GPX4^flox/+^: F1: 5′-CTGGTAGCATATTCTAGGGGTGT-3′; R1: 5′-GAAGAATCCGGTGCCAAAGAAAG-3′. The PCR primers for S100a8-Cre-EGFP were as follows: F1: 5′-CATCTGCTGGTTTGGTTATTTGGAG-3′; R1: 5′-CTTGCGAACCTCATCACTCGTTG-3′. The genotypes of the mice were then determined through nucleic acid agarose gel electrophoresis. A 200-bp band indicates a GPX4 homozygote, a 131-bp band indicates wild type, and the presence of both 300- and 200-bp bands indicates a GPX4 heterozygote (Fig. S1A). The presence of a 277-bp band indicates S100a8-Cre (Fig. S1B).

First, F_1_ heterozygous GPX4 ^flox/+^ mice were bred to produce F_2_ homozygous Gpx4^flox/flox^ mice. The F_2_ generation was then mated with S100a8-Cre to obtain the F_3_ generation, and finally, F_3_ was mated with F_2_ to produce the F_4_ generation of neutrophil-specific GPX4 knockout mice (Fig. S1C). Western blot analysis verified a marked decrease in GPX4 protein levels in blood neutrophils obtained from GPX4-cko compared to those in GPX4 f/f (Fig. S1D and E).

### Preparation of CSE

The preparation of CSE was based on our team’s previous method [55]. Briefly, CS was extracted using a peristaltic pump, and the smoke from one cigarette was passed through 10 ml of RPMI 1640 medium to generate 10% CSE.

### Cell treatment and cell viability

Various stimuli were applied to the cells, with the concentrations used and references listed below: CSE, 2% [11]; RSL3, 0.4 μM; LY294002, 10 μM [56]; DNase I, 0.1 mg/ml [11]; and Dex, 1 μM [57]. The cells were plated at a density of 5 × 10^5^ cells per well in a 96-well plate and subjected to various treatments. After 8 h, cell viability was assessed using a Cell Counting Kit-8 assay.

### Calcein/PI cell viability/cytotoxicity staining

Neutrophils isolated from the peripheral blood of healthy individuals were divided into 2 groups: the control group and the 2% CSE stimulation group. Neutrophils (2 × 10^6^ cells per well) were seeded onto poly-l-lysine-coated coverslips and placed in a 24-well plate. Calcein/PI staining (diluted 1,000-fold) was added to each well to assess cell survival and death in the 2 groups at 8, 16, and 24 h post-isolation.

### SYTOX Green staining

Neutrophils (2 × 10^6^ cells per well) isolated from healthy subjects, as well as GC-sensitive and GC-resistant COPD patients, were stimulated with PMA at a final concentration of 30 ng/ml, seeded onto poly-l-lysine-coated coverslips, and placed in a 24-well plate. Following 8-h incubation, 1 μM SYTOX Green was added to observe double-stranded DNA levels. Fluorescence was recorded with excitation wavelengths set at 502 nm and emission wavelengths at 525 nm.

### ROS levels

Neutrophils (2 × 10^6^ cells per well) were seeded onto poly-l-lysine-coated coverslips and placed in a 24-well plate, incubated at 37 °C with RPMI 1640 medium. After 16 h, 0.5 μM DCFH-DA was added to each well. ROS levels were observed with a fluorescence microscope.

### Lipid peroxidation detection

Neutrophils (1 × 10^7^ cells per well) were stimulated with RSL3 (0.4 μM), treated with or without 2% CSE and cultured in 20-mm poly-l-lysine-coated glass-bottom dishes for 8 h. The level of lipid peroxidation was detected by a Liperfluo staining assay kit. The cells were incubated with the probe Liperfluo (1 μM) at 37 °C for 30 min, followed by 3 washes with PBS. Lipid peroxidation levels were assessed using confocal microscopy (Olympus FV3000, Japan).

### FerroOrange staining

Neutrophils (1 × 10^7^ cells per well) were stimulated with RSL3 (0.4 μM), treated with or without 2% CSE and cultured in 20-mm poly-l-lysine-coated glass-bottom dishes for 8 h. The levels of Fe^2+^ in the cells were assessed via a FerroOrange staining assay kit. The cells were incubated with the probe FerroOrange (1 μM) at 37 °C for 30 min. Fe^2+^ levels were assessed using confocal microscopy (Olympus FV3000, Japan).

### Immunofluorescence staining of cells

Neutrophils (2 × 10^6^ cells per well) were seeded onto poly-l-lysine-coated coverslips and placed in a 24-well plate. The cells were treated with different stimuli (30 ng/ml PMA, 1% CSE, or 2% CSE). After 8 h, immunofluorescence staining was performed. The immunofluorescence staining procedure was as follows: First, the cells were fixed with 4% paraformaldehyde fix solution at 4 °C for 20 min, followed by permeabilization with 0.5% Triton X-100 at room temperature for 10 min. The cells were then blocked with 5% bovine serum albumin at room temperature for 30 min. Afterward, 200 μl of a 1:100 dilution of anti-CitH3 primary antibody was added, and the cells were incubated at 4 °C overnight. On the following day, the corresponding secondary antibody was added and incubated at room temperature for 1 h. Finally, DAPI was used to stain the nuclei for 15 min, and images were taken using a fluorescence microscope.

### Single-cell RNA sequencing analysis

Neutrophils obtained from healthy donors were randomly assigned to either a control group or a 2% CSE-exposed group (*n* = 3 per group). Following 8 h of treatment, cells were harvested for transcriptomic profiling. RNA extraction was performed using the TRIzol reagent kit (ABclonal, Wuhan, China), with subsequent sequencing conducted on the NovelCyto v1.0 platform. Quality control of raw sequencing data was performed using FAST-QC, which evaluated nucleotide distribution, sequencing quality metrics, GC content, and *k*-mer frequency. Gene expression levels were quantified using reads per kilobase per million mapped reads). Differentially expressed genes were defined as those exhibiting a fold change >1.5 with a false discovery rate (FDR)-adjusted *P* value <0.05. Visualization of gene expression patterns was achieved through the generation of volcano plots and heatmaps based on fragments per kilobase per million values. Functional enrichment analysis was conducted to identify significantly altered pathways, with statistical significance determined by Fisher’s exact test (FDR <0.05 considered significant).

### Quantitative real-time PCR

Neutrophil total RNA was isolated using an RNA extraction kit (Baidai Biotechnology, Changzhou, China). RNA quality was verified by measuring the 260/280 ratio on a NanoDrop spectrophotometer. For complementary DNA synthesis, 1 μg of RNA was reverse-transcribed using an All-in-One RT system (ToloBio, Shanghai, China). quantitative reverse transcription PCR was then performed with a Q3 SYBR qPCR master mix (ToloBio) on a LightCycler 96 system (Roche). The level of each transcript was normalized to the β-actin transcript level and was calculated using the 2^−∆Ct^ method (∆Ct = Ct_(gene of interest)_ − Ct_β-actin_). Primer sequences are listed in Table S2.

### Western blotting analysis

Total protein was extracted from lung tissues or cells using radioimmunoprecipitation assay lysis buffer. Protein concentrations were determined by bicinchoninic acid assay, and equal amounts (20 μg per lane) were resolved by 12.5% sodium dodecyl sulfate–polyacrylamide gel electrophoresis and transferred to polyvinylidene difluoride membranes. After blocking, membranes were probed overnight at 4 °C with primary antibodies. Following PBS with Tween 20 washes, horseradish peroxidase-conjugated secondary antibodies were applied. Protein bands were visualized using enhanced chemiluminescence, with β-actin as the loading control. Band intensities were quantified using the ImageJ software.

### Statistics

Data are represented as mean ± standard deviation (SD). For comparisons involving 2 groups, the normality of data was checked using the Shapiro–Wilk test; datasets that followed a normal distribution were evaluated using *t* tests, while nonnormal datasets were analyzed using Mann–Whitney *U* tests. For comparisons involving multiple groups, one-way analysis of variance, followed by Tukey’s post hoc test, was employed. Pearson’s correlation tests were conducted for correlation analysis. *P* < 0.05 indicates a significant difference. We used GraphPad Prism 8 for statistical analysis.

## Ethical Approval

All listed authors consent to the submission, and all data are used with the consent of the person generating the data.

## Data Availability

The data that support the findings of this study are available from the corresponding authors upon reasonable request.

## References

[1] Global Initiative for Chronic Obstructive Lung Disease. Global strategy for the diagnosis, management, and prevention of COPD. Global Initiative for Chronic Obstructive Lung Disease. 2024. [accessed 13 Nov 2023] http://goldcopd.org/

[2] López-Campos JL, Quintana Gallego E, Carrasco Hernández L. Status of and strategies for improving adherence to COPD treatment. Int J Chron Obstruct Pulmon Dis. 2019;14:1503–1515. 31371936 10.2147/COPD.S170848 PMC6628097

[3] Reddy AT, Lakshmi SP, Banno A, Reddy RC. Glucocorticoid receptor α mediates roflumilast’s ability to restore dexamethasone sensitivity in COPD. Int J Chron Obstruct Pulmon Dis. 2020;15:125–134. 32021151 10.2147/COPD.S230188 PMC6969699

[4] Liu Q, Hua L, Bao C, Kong L, Hu J, Liu C, Li Z, Xu S, Liu X. Inhibition of spleen tyrosine kinase restores glucocorticoid sensitivity to improve steroid-resistant asthma. Front Pharmacol. 2022;13: Article 885053. 35600871 10.3389/fphar.2022.885053 PMC9117698

[5] Zhou L, Roth M, Papakonstantinou E, Tamm M, Stolz D. Expression of glucocorticoid receptor and HDACs in airway smooth muscle cells is associated with response to steroids in COPD. Respir Res. 2024;25(1):227. 38812021 10.1186/s12931-024-02769-3 PMC11137987

[6] Anzalone G, Gagliardo R, Bucchieri F, Albano GD, Siena L, Montalbano AM, Bonanno A, Riccobono L, Pieper MP, Gjomarkaj M, et al. IL-17A induces chromatin remodeling promoting IL-8 release in bronchial epithelial cells: Effect of tiotropium. Life Sci. 2016;152:107–116. 27038884 10.1016/j.lfs.2016.03.031

[7] Dong J, Chen J, Li Q, Qiu S. Knockdown of FKBP3 suppresses nasopharyngeal carcinoma cell growth, invasion and migration, deactivated NF-κB/IL-6 signaling pathway through inhibiting histone deacetylase 2 expression. Chin J Physiol. 2023;66(2):85–92. 37082996 10.4103/cjop.CJOP-D-22-00075

[8] Wei P, Huang Z, Gan L, Li Y, Qin C, Liu G. Nintedanib ameliorates tracheal stenosis by activating HDAC2 and suppressing IL-8 and VEGF in rabbit. Am J Transl Res. 2020;12(8):4739–4748. 32913546 PMC7476127

[9] Barnes PJ. Inhaled corticosteroids in COPD: A controversy. Respiration. 2010;80(2):89–95. 20501985 10.1159/000315416

[10] Jiang Z, Zhu L. Update on molecular mechanisms of corticosteroid resistance in chronic obstructive pulmonary disease. Pulm Pharmacol Ther. 2016;37:1–8. 26805715 10.1016/j.pupt.2016.01.002

[11] Chen J, Wang T, Li X, Gao L, Wang K, Cheng M, Zeng Z, Chen L, Shen Y, Wen F. DNA of neutrophil extracellular traps promote NF-κB-dependent autoimmunity via cGAS/TLR9 in chronic obstructive pulmonary disease. Signal Transduct Target Ther. 2024;9(1):163. 38880789 10.1038/s41392-024-01881-6 PMC11180664

[12] Zhang H, Qiu SL, Tang QY, Zhou X, Zhang JQ, He ZY, Bai J, Li MH, Deng JM, Liang Y, et al. Erythromycin suppresses neutrophil extracellular traps in smoking-related chronic pulmonary inflammation. Cell Death Dis. 2019;10(9):678. 31515489 10.1038/s41419-019-1909-2 PMC6742640

[13] Hosseinzadeh A, Thompson PR, Segal BH, Urban CF. Nicotine induces neutrophil extracellular traps. J Leukoc Biol. 2016;100(5):1105–1112. 27312847 10.1189/jlb.3AB0815-379RR PMC5069087

[14] Liu X, Li T, Chen H, Yuan L, Ao H. Role and intervention of PAD4 in NETs in acute respiratory distress syndrome. Respir Res. 2024;25(1):63. 38291476 10.1186/s12931-024-02676-7 PMC10829387

[15] Zhou Y, Xu L, Jin P, Li N, Chen X, Yang A, Qi H. NET-targeted nanoparticles for antithrombotic therapy in pregnancy. iScience. 2024;27(5): Article 109823. 38756418 10.1016/j.isci.2024.109823 PMC11097077

[16] Masuda S, Nakazawa D, Shida H, Miyoshi A, Kusunoki Y, Tomaru U, Ishizu A. NETosis markers: Quest for specific, objective, and quantitative markers. Clin Chim Acta. 2016;459:89–93. 27259468 10.1016/j.cca.2016.05.029

[17] Peng X, Li Y, Zhao W, Yang S, Huang J, Chen Y, Wang Y, Gong Z, Chen X, Yu C, et al. Blockade of neutrophil extracellular traps ameliorates toluene diisocyanate-induced steroid-resistant asthma. Int Immunopharmacol. 2023;117: Article 109719. 36827917 10.1016/j.intimp.2023.109719

[18] Allam V, Pavlidis S, Liu G, Kermani NZ, Simpson J, To J, Donnelly S, Guo YK, Hansbro PM, Phipps S, et al. Macrophage migration inhibitory factor promotes glucocorticoid resistance of neutrophilic inflammation in a murine model of severe asthma. Thorax. 2023;78(7):661–673. 36344253 10.1136/thorax-2021-218555

[19] Thiam HR, Wong SL, Wagner DD, Waterman CM. Cellular mechanisms of NETosis. Annu Rev Cell Dev Biol. 2020;36:191–218. 32663035 10.1146/annurev-cellbio-020520-111016 PMC8499668

[20] Yipp BG, Kubes P. NETosis: How vital is it? Blood. 2013;122(16):2784–2794. 24009232 10.1182/blood-2013-04-457671

[21] Chen Y, Hu H, Tan S, Dong Q, Fan X, Wang Y, Zhang H, He J. The role of neutrophil extracellular traps in cancer progression, metastasis and therapy. Exp Hematol Oncol. 2022;11(1):99. 36384979 10.1186/s40164-022-00345-3 PMC9667637

[22] Aspera-Werz RH, Mück J, Linnemann C, Herbst M, Ihle C, Histing T, Nussler AK, Ehnert S. Nicotine and cotinine induce neutrophil extracellular trap formation—Potential risk for impaired wound healing in smokers. Antioxidants. 2022;11(12): Article 2424. 36552632 10.3390/antiox11122424 PMC9774423

[23] Matthews JB, Chen FM, Milward MR, Wright HJ, Carter K, McDonagh A, Chapple IL. Effect of nicotine, cotinine and cigarette smoke extract on the neutrophil respiratory burst. J Clin Periodontol. 2011;38(3):208–218. 21214612 10.1111/j.1600-051X.2010.01676.x

[24] Brembach TC, Sabat R, Witte K, Schwerdtle T, Wolk K. Molecular and functional changes in neutrophilic granulocytes induced by nicotine: A systematic review and critical evaluation. Front Immunol. 2023;14:1281685. 38077313 10.3389/fimmu.2023.1281685 PMC10702484

[25] Ng MSF, Kwok I, Tan L, Shi C, Cerezo-Wallis D, Tan Y, Leong K, Calvo GF, Yang K, Zhang Y, et al. Deterministic reprogramming of neutrophils within tumors. Science. 2024;383(6679):eadf6493. 38207030 10.1126/science.adf6493 PMC11087151

[26] Zeng Z, Li T, Liu X, Ma Y, Luo L, Wang Z, Zhao Z, Li H, He X, Zeng H, et al. DNA dioxygenases TET2 deficiency promotes cigarette smoke induced chronic obstructive pulmonary disease by inducing ferroptosis of lung epithelial cell. Redox Biol. 2023;67: Article 102916. 37812881 10.1016/j.redox.2023.102916 PMC10579541

[27] Liu Y, Wan Y, Jiang Y, Zhang L, Cheng W. GPX4: The hub of lipid oxidation, ferroptosis, disease and treatment. Biochim Biophys Acta Rev Cancer. 2023;1878(3): Article 188890. 37001616 10.1016/j.bbcan.2023.188890

[28] Dai E, Chen X, Linkermann A, Jiang X, Kang R, Kagan VE, Bayir H, Yang WS, Garcia-Saez AJ, Ioannou MS, et al. A guideline on the molecular ecosystem regulating ferroptosis. Nat Cell Biol. 2024;26(9):1447–1457. 38424270 10.1038/s41556-024-01360-8 PMC11650678

[29] Zhao Y, Liu Z, Liu G, Zhang Y, Liu S, Gan D, Chang W, Peng X, Sung ES, Gilbert K, et al. Neutrophils resist ferroptosis and promote breast cancer metastasis through aconitate decarboxylase 1. Cell Metab. 2023;35(10):1688–1703.e10. 37793345 10.1016/j.cmet.2023.09.004 PMC10558089

[30] Tu H, Ren H, Jiang J, Shao C, Shi Y, Li P. Dying to defend: Neutrophil death pathways and their implications in immunity. Adv Sci. 2024;11(8):e2306457. 10.1002/advs.202306457 PMC10885667 38044275

[31] Zhang W, Lei Y, Zhang T, You B, Zhang J, Zhou Y, Zhang S, Li X, Liu Y, Shen L, et al. IL-8 promotes pyroptosis through ERK pathway and mediates glucocorticoid resistance in chronic rhinosinusitis with nasal polyps. Inflamm Res. 2025;74(1):20. 39821406 10.1007/s00011-024-01982-6

[32] Nabe T. Steroid-resistant asthma and neutrophils. Biol Pharm Bull. 2020;43(1):31–35. 31902928 10.1248/bpb.b19-00095

[33] Hou T, Zhu L, Zhang Y, Tang Y, Gao Y, Hua S, Ci X, Peng L. Lipid peroxidation triggered by the degradation of xCT contributes to gasdermin D-mediated pyroptosis in COPD. Redox Biol. 2024;77: Article 103388. 39374556 10.1016/j.redox.2024.103388 PMC11491731

[34] Dong T, Fan X, Zheng N, Yan K, Hou T, Peng L, Ci X. Activation of Nrf2 signalling pathway by tectoridin protects against ferroptosis in particulate matter-induced lung injury. Br J Pharmacol. 2023;180(19):2532–2549. 37005797 10.1111/bph.16085

[35] Zhu L, Zhang Q, Hua C, Ci X. Melatonin alleviates particulate matter-induced liver fibrosis by inhibiting ROS-mediated mitophagy and inflammation via Nrf2 activation. Ecotoxicol Environ Saf. 2023;268: Article 115717. 37992643 10.1016/j.ecoenv.2023.115717

[36] Zhang H, Wu D, Wang Y, Shi Y, Shao Y, Zeng F, Spencer CB, Ortoga L, Wu D, Miao C. Ferritin-mediated neutrophil extracellular traps formation and cytokine storm via macrophage scavenger receptor in sepsis-associated lung injury. Cell Commun Signal. 2024;22(1):97. 38308264 10.1186/s12964-023-01440-6 PMC10837893

[37] Jia J, Wang M, Meng J, Ma Y, Wang Y, Miao N, Teng J, Zhu D, Shi H, Sun Y, et al. Ferritin triggers neutrophil extracellular trap-mediated cytokine storm through Msr1 contributing to adult-onset Still’s disease pathogenesis. Nat Commun. 2022;13(1):6804. 36357401 10.1038/s41467-022-34560-7 PMC9648446

[38] Makrygiannakis D, Revu S, Engström M, af Klint E, Nicholas AP, Pruijn GJ, Catrina AI. Local administration of glucocorticoids decreases synovial citrullination in rheumatoid arthritis. Arthritis Res Ther. 2012;14(1):R20. 22284820 10.1186/ar3702 PMC3392813

[39] Lea S, Higham A, Beech A, Singh D. How inhaled corticosteroids target inflammation in COPD. Eur Respir Rev. 2023;32(170): Article 230084. 37852657 10.1183/16000617.0084-2023 PMC10582931

[40] Mutua V, Gershwin LJ. A review of neutrophil extracellular traps (NETs) in disease: Potential anti-NETs therapeutics. Clin Rev Allergy Immunol. 2021;61(2):194–211. 32740860 10.1007/s12016-020-08804-7 PMC7395212

[41] Lee KH, Lee J, Jeong J, Woo J, Lee CH, Yoo CG. Cigarette smoke extract enhances neutrophil elastase-induced IL-8 production via proteinase-activated receptor-2 upregulation in human bronchial epithelial cells. Exp Mol Med. 2018;50(7):1–9. 10.1038/s12276-018-0114-1 PMC6035212 29980681

[42] Hudock KM, Collins MS, Imbrogno M, Snowball J, Kramer EL, Brewington JJ, Gollomp K, McCarthy C, Ostmann AJ, Kopras EJ, et al. Neutrophil extracellular traps activate IL-8 and IL-1 expression in human bronchial epithelia. Am J Physiol Lung Cell Mol Physiol. 2020;319(1):L137–l147. 32159969 10.1152/ajplung.00144.2019 PMC7468846

[43] Zha C, Meng X, Li L, Mi S, Qian D, Li Z, Wu P, Hu S, Zhao S, Cai J, et al. Neutrophil extracellular traps mediate the crosstalk between glioma progression and the tumor microenvironment via the HMGB1/RAGE/IL-8 axis. Cancer Biol Med. 2020;17(1):154–168. 32296583 10.20892/j.issn.2095-3941.2019.0353 PMC7142852

[44] Jiang Y, Liu B, Bao X, Zhou P, Li J. TNF-α regulates the glucocorticoid receptor alpha expression in human nasal epithelial cells via p65-NF-κb and p38-MAPK signaling pathways. Iran J Biotechnol. 2023;21(1): Article e3117. 36811108 10.30498/ijb.2022.298590.3117 PMC9938934

[45] Yamasaki A, Okazaki R, Harada T. Neutrophils and asthma. Diagnostics. 2022;12(5):1175. 35626330 10.3390/diagnostics12051175 PMC9140072

[46] Bansal A, Kooi C, Kalyanaraman K, Gill S, Thorne A, Chandramohan P, Necker-Brown A, Mostafa MM, Milani A, Leigh R, et al. Synergy between interleukin-1β, interferon-γ, and glucocorticoids to induce TLR2 expression involves NF-κB, STAT1, and the glucocorticoid receptor. Mol Pharmacol. 2023;105(1):23–38. 37863662 10.1124/molpharm.123.000740

[47] Deng F, Li X, Tang C, Chen J, Fan B, Liang J, Zhen X, Tao R, Zhang S, Cong Z, et al. Mechanisms of Xiong-Pi-Fang in treating coronary heart disease associated with depression: A systematic pharmacology strategy and *in vivo* pharmacological validation. J Ethnopharmacol. 2022;298: Article 115631. 35987411 10.1016/j.jep.2022.115631

[48] Yang C, Wang Z, Li L, Zhang Z, Jin X, Wu P, Sun S, Pan J, Su K, Jia F, et al. Aged neutrophils form mitochondria-dependent vital NETs to promote breast cancer lung metastasis. J Immunother Cancer. 2021;9(10): Article e002875. 34716206 10.1136/jitc-2021-002875 PMC8559246

[49] Wang K, Wang M, Liao X, Gao S, Hua J, Wu X, Guo Q, Xu W, Sun J, He Y, et al. Locally organised and activated Fth1^hi^ neutrophils aggravate inflammation of acute lung injury in an IL-10-dependent manner. Nat Commun. 2022;13(1):7703. 36513690 10.1038/s41467-022-35492-y PMC9745290

[50] Ruscitti P, Berardicurti O, Barile A, Cipriani P, Shoenfeld Y, Iagnocco A, Giacomelli R. Severe COVID-19 and related hyperferritinaemia: More than an innocent bystander? Ann Rheum Dis. 2020;79(11):1515–1516. 32434816 10.1136/annrheumdis-2020-217618

[51] Wang Y, Shen Z, Zhao S, Huang D, Wang X, Wu Y, Pei C, Shi S, Jia N, He Y, et al. Sipeimine ameliorates PM2.5-induced lung injury by inhibiting ferroptosis via the PI3K/Akt/Nrf2 pathway: A network pharmacology approach. Ecotoxicol Environ Saf. 2022;239: Article 113615. 35567927 10.1016/j.ecoenv.2022.113615

[52] Fan JB, Yuan K, Zhu XH, Cui SY, Yi H, Zhang W. Neuroligin-3 activates Akt-dependent Nrf2 cascade to protect osteoblasts from oxidative stress. Free Radic Biol Med. 2023;208:807–819. 37774803 10.1016/j.freeradbiomed.2023.09.032

[53] Zhang H, Pan J, Huang S, Chen X, Chang ACY, Wang C, Zhang J, Zhang H. Hydrogen sulfide protects cardiomyocytes from doxorubicin-induced ferroptosis through the SLC7A11/GSH/GPx4 pathway by Keap1 S-sulfhydration and Nrf2 activation. Redox Biol. 2024;70: Article 103066. 38359744 10.1016/j.redox.2024.103066 PMC10877437

[54] Amaral EP, Foreman TW, Namasivayam S, Hilligan KL, Kauffman KD, Barbosa Bomfim CC, Costa DL, Barreto-Duarte B, Gurgel-Rocha C, Santana MF, et al. GPX4 regulates cellular necrosis and host resistance in *Mycobacterium tuberculosis* infection. J Exp Med. 2022;219(11): Article e20220504. 36069923 10.1084/jem.20220504 PMC9458471

[55] Fan X, Dong T, Yan K, Ci X, Peng L. PM2.5 increases susceptibility to acute exacerbation of COPD via NOX4/Nrf2 redox imbalance-mediated mitophagy. Redox Biol. 2023;59: Article 102587. 36608590 10.1016/j.redox.2022.102587 PMC9813701

[56] Martelli AM, Tazzari PL, Tabellini G, Bortul R, Billi AM, Manzoli L, Ruggeri A, Conte R, Cocco L. A new selective AKT pharmacological inhibitor reduces resistance to chemotherapeutic drugs, TRAIL, all-*trans*-retinoic acid, and ionizing radiation of human leukemia cells. Leukemia. 2003;17(9):1794–1805. 12970779 10.1038/sj.leu.2403044

[57] Ouyang S, Liu C, Xiao J, Chen X, Lui AC, Li X. Targeting IL-17A/glucocorticoid synergy to CSF3 expression in neutrophilic airway diseases. JCI Insight. 2020;5(3): Article e132836. 32051346 10.1172/jci.insight.132836 PMC7098787

