# Supplementary material for: Long-Term Cigarette Smoke Exposure Promotes Neutrophil Ferroptosis Resistance, Inducing Neutrophil Extracellular Trap Formation and Driving Glucocorticoid Resistance in Chronic Obstructive Pulmonary Disease
Source: Research (Wash D C). 2025 Jul 15;8:0751. doi: 10.34133/research.0751 (PMC12260225; doi:10.34133/research.0751)
Supplement: Supplementary 1 — Tables S1 and S2 Figs. S1 to S4 [file research.0751.f1.zip › Supplementary materials.docx]

**Supplementary materials**

**Supplementary Table 1 Basic information of clinical samples**

|  | Healthy subjects  (n=10) | COPD group | |  |
| --- | --- | --- | --- | --- |
|  |  | GC-sensitive （n=4） | GC- resistant (n=6) | *P* |
| Age | 60±8 | 61±15 | 65±5 | NS |
| Male n (%) | 5 (50%) | 0 (0%) | 6 (100%) | * |
| Smoker n（%） | 0(0%) | 0 (0%) | 6 (100%) | * |
| CAT before treatment | NA | 26±5 | 28±5 | NS |
| CAT after treatment | NA | 7±2 | 21±5 | * |
| WBC (×10^9^/L) | NA | 6.2±0.43 | 7.4±3.3 | NS |
| Neutrophil (×10^9^/L) | NA | 4.0±1.0 | 5.6±3.3 | NS |
| Eosinophil (×10^9^/L) | NA | 0.22±0.19 | 0.16±0.1 | NS |
| FEV1% pred | NA | 41±9 | 27±8 | * |
| NE (ng/ml) | NA | 61.05±15.51 | 86.73±19 | * |
| IL-8 (pg/ml) | NA | 63.5±35.51 | 203.3±26.52 | * |

Abbreviation: GC: Glucocorticoid; CAT: COPD Assessment Test; WBC: White blood cell; FEV1% pred: Forced expiratory volume in one second predicted values; NE: Neutrophil elastase; NA: Not available.* *P*＜0.05 GC- sensitive compared with GC-resistant group.NS:No significance.

**Supplementary Table 2 Primer sequences for various genes in human neutrophils**

| **Gene** | **Primer sequences** （5’-3’） |  |
| --- | --- | --- |
| β-Actin | TTCCTGGGCATGGAGTCCT AGGAGGAGCAATGATCTTGATC | 204bp |
| Nrf2 | GGATCTGCCAACTACTCCCAG CGTAGCCGAAGAAACCTCATTG | 182bp |
| SLC7A11 | TCTCCAAAGGAGGTTACCTGC AGACTCCCCTCAGTAAAGTGAC | 123bp |
| GPX4 | ACAAGAACGGCTGCGTGGTGAA GCCACACACTTGTGGAGCTAGA | 100bp |
| GCLM | CGCACAGCGAGGAGGAGTTT AATCCAGCTGTGCAACTCCAA | 192bp |
| NQO1 | AGGACCCTTCCGGAGTAAGA CCACTCTGAATTGGCCAGAG | 120bp |
| HMOX1 | CCACTCTGAATTGGCCAGAG CCACGGGGGCAGAATCTTG | 138bp |
| FTH1 | CCACGGGGGCAGAATCTTG  GAAGGAAGATTCGGCCACCT | 156bp |
| FTL | TACACCTACCTCTCTCTGGGC  TCTTCAGCTGGCTTCTTGATG | 179bp |
| GCLC | TAACGTGACGCTTGAAGAGG  AGTTTGGAGGAGGGGGCTTA | 182bp |

**Supplementary Figure Legends**

Supplementary figure 1 Identification and breeding of mice with neutrophil-specific GPX4 deletion. (A) 1,5, and 6 are wild type mice; 2 and 4 are GPX4 ^flox/flox^ mice; 3,7, and 8 are GPX4 ^flox/+^ mice. (B) 5 represents a wild type mice; 1,2,3,4,6,7,and 8 are S100a8-Cre mice. (C) Breeding strategy for mice with neutrophil-specific GPX4 deletion. (D,E) Western blot analysis of GPX4 expression in neutrophils from GPX4 flox/flox and GPX4-cko mice. Statistical analysis: The data are presented as the means ± SDs; Differences were assessed via t-tests. *P* < 0.05 indicated a significant difference. * *P*＜0.05 compared with the GPX4 ^flox/flox^ mice .

Supplementary figure 2 CSE inhibited neutrophil death. Neutrophils isolated from the peripheral blood of healthy individuals were divided into two groups: the control group and the 2% CSE stimulation group. Calcein/PI staining was used to assess cell survival and death in both groups at 8, 16, and 24 hours post-isolation. In the control group, the number of surviving cells gradually decreased while the number of dead cells increased over time; in contrast, CSE stimulation inhibited neutrophil death and increased cell viability (scale bar, 20 µm).

Supplementary figure 3 As neutrophil activity increased, the levels of inflammatory factor and the release of NETs also increased. Neutrophils isolated from the peripheral blood of healthy individuals were exposed to different concentrations of CSE. (A,B) CSE elevated the levels of IL-8 and NE in a dose-dependent manner in the culture medium measured by ELISA. (C,D) With the increase in CSE concentration, the expression of PADI4 and CitH3 in neutrophils increased. Meanwhile, the expression of HDAC2 decreased. Statistical analysis: The data are presented as the means ± SDs. Differences were assessed via one-way ANOVA, followed by Tukey's post hoc test for significance. *P* < 0.05 indicated a significant difference. * *P*＜0.05 compared with the control group.

Supplementary figure 4 There were significant differences among the groups of transcriptome sequencing data, and with good reproducibility. (A) Results of FPKM expression distribution. (B) Results of Sample Correlation. (C) Results of Sample PCA.
