# Supplementary figures and images for: Long-Term Cigarette Smoke Exposure Promotes Neutrophil Ferroptosis Resistance, Inducing Neutrophil Extracellular Trap Formation and Driving Glucocorticoid Resistance in Chronic Obstructive Pulmonary Disease
Source: Research (Wash D C). 2025 Jul 15;8:0751. doi: 10.34133/research.0751 (PMC12260225; doi:10.34133/research.0751)

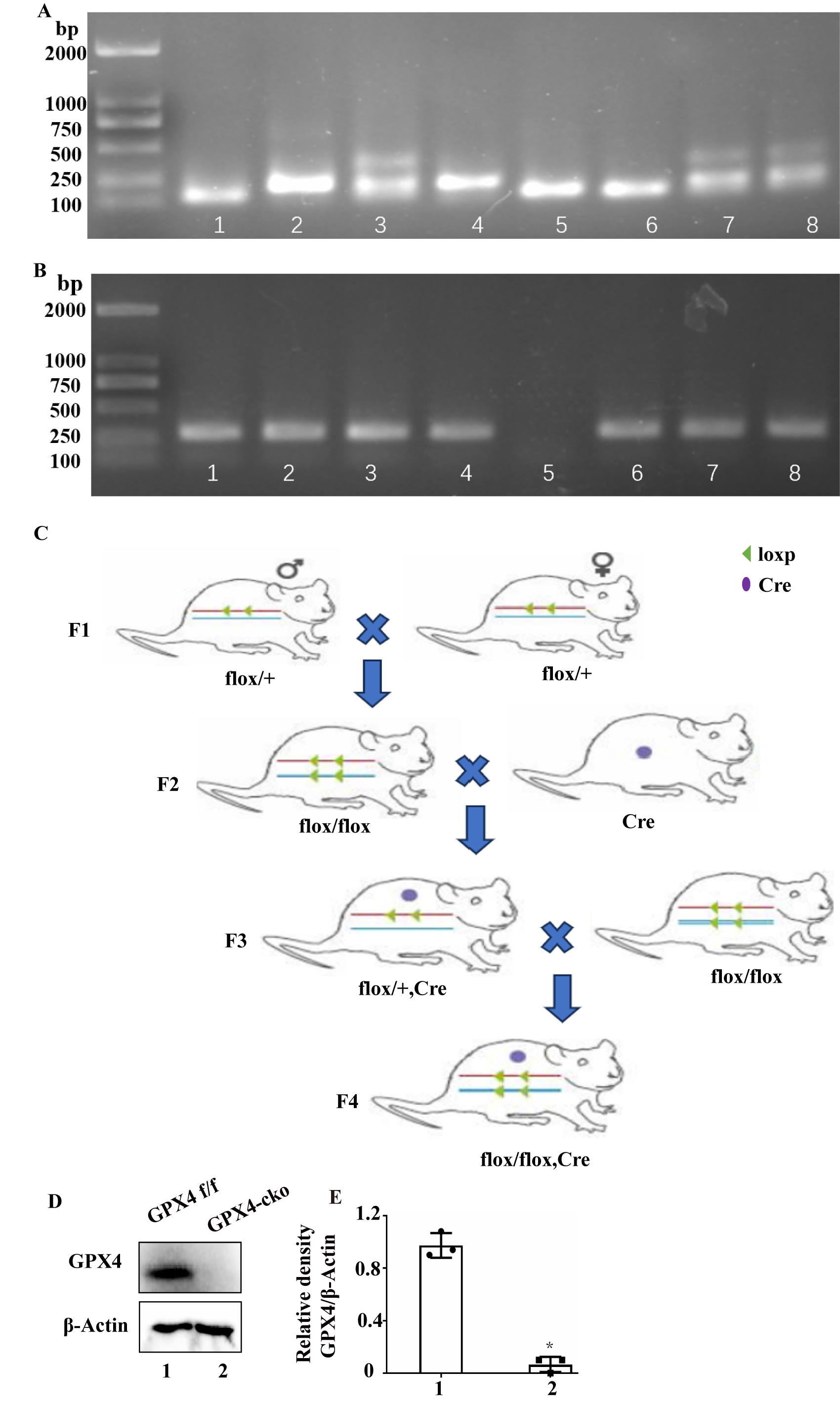

Supplement: Supplementary 1 — Tables S1 and S2 Figs. S1 to S4 [file research.0751.f1.zip › supplementary fig.1.tif]

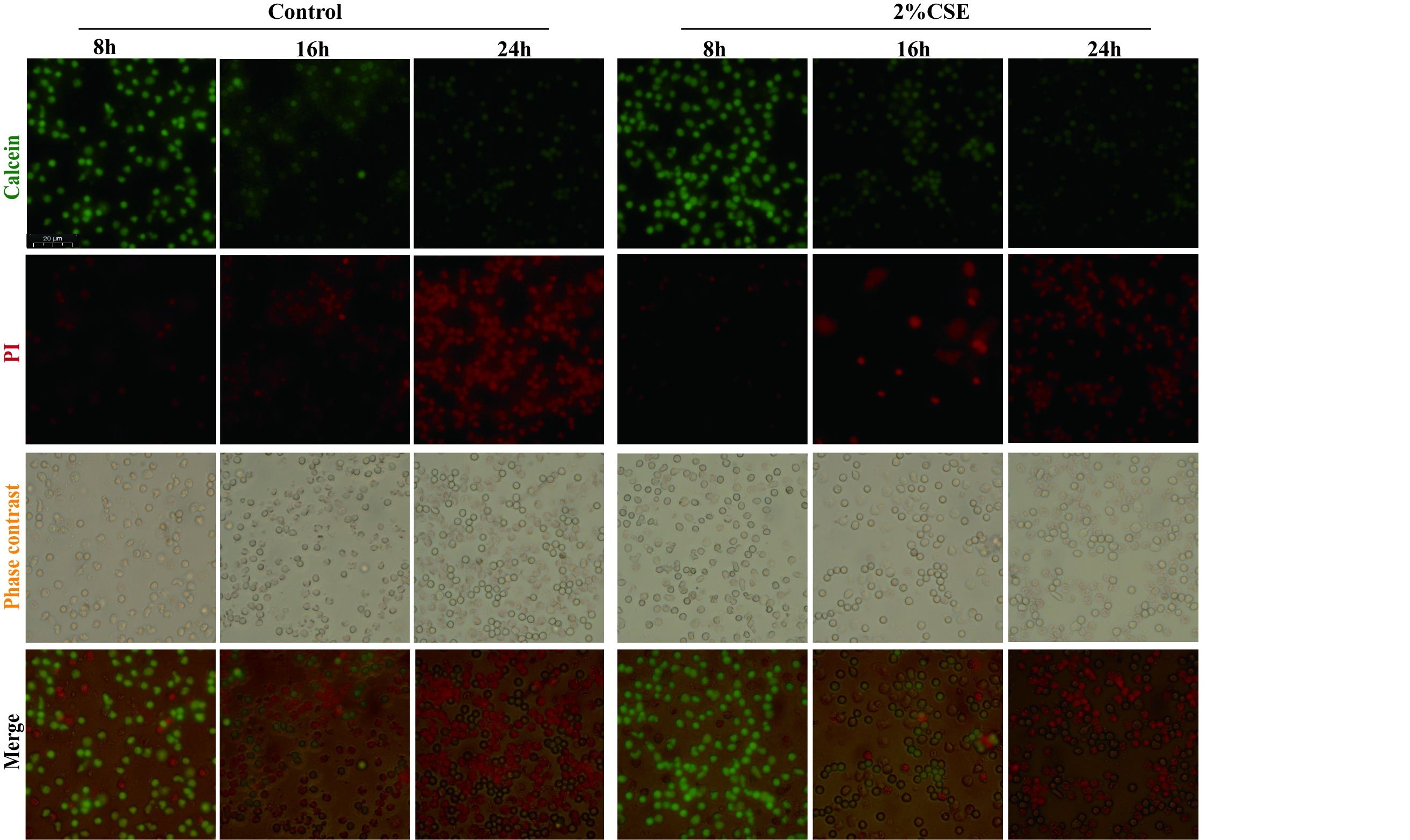

Supplement: Supplementary 1 — Tables S1 and S2 Figs. S1 to S4 [file research.0751.f1.zip › supplementary fig.2.tif]

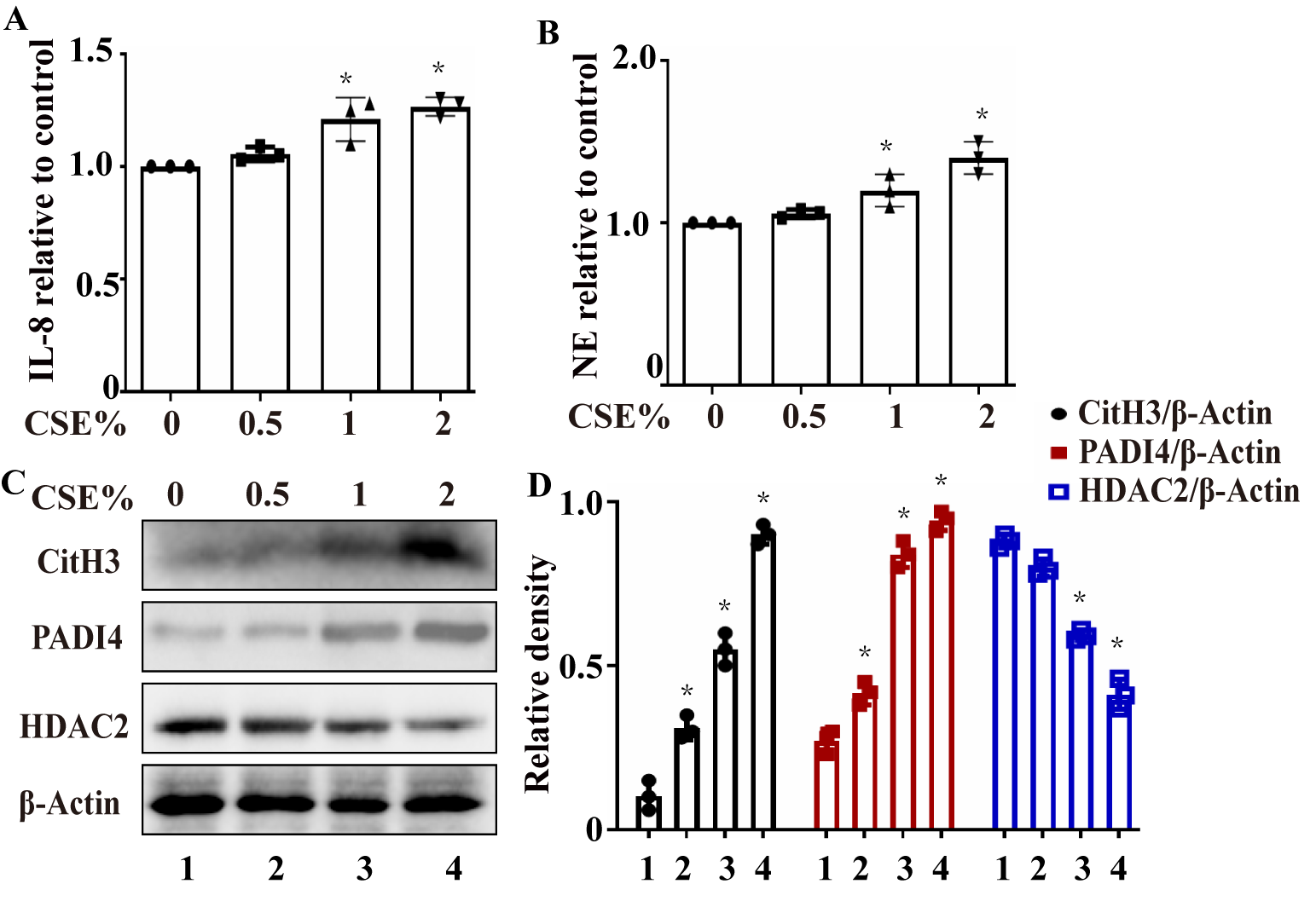

Supplement: Supplementary 1 — Tables S1 and S2 Figs. S1 to S4 [file research.0751.f1.zip › supplementary fig.3.tif]

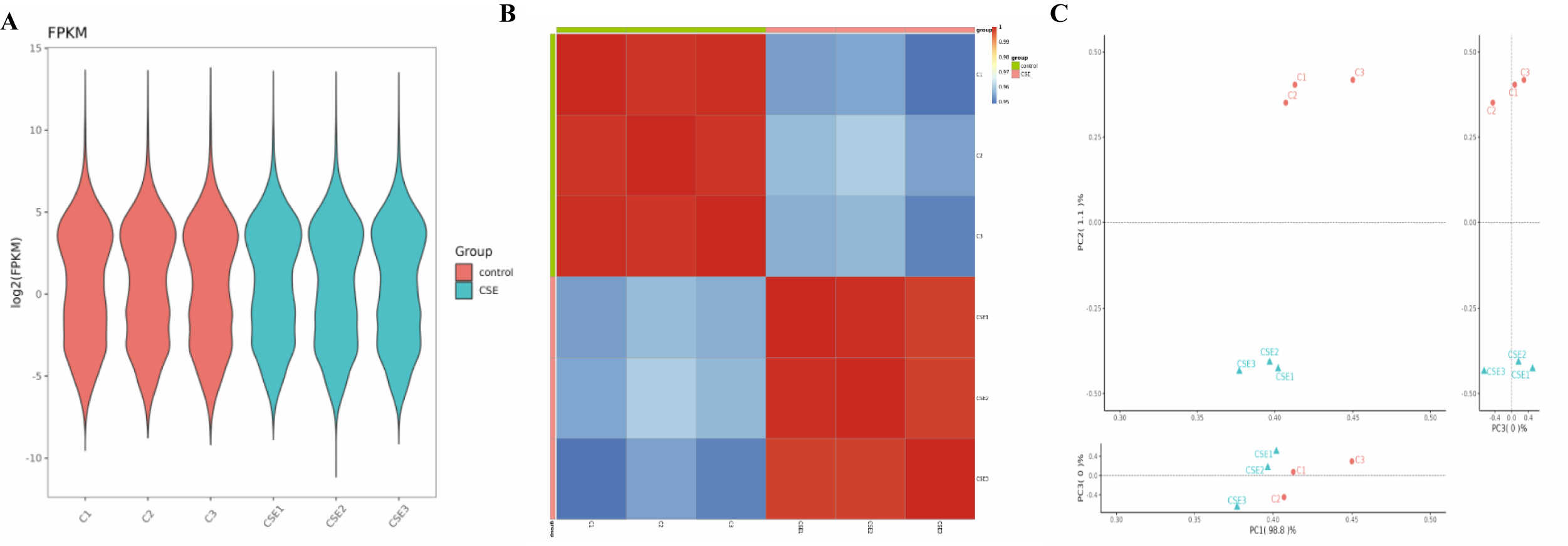

Supplement: Supplementary 1 — Tables S1 and S2 Figs. S1 to S4 [file research.0751.f1.zip › supplementary fig.4.tif]
